# Enhanced stability of M1 protein mediated by a phospho-resistant mutation promotes the replication of prevailing avian influenza virus in mammals

**DOI:** 10.1371/journal.ppat.1010645

**Published:** 2022-07-06

**Authors:** Chenxi Wang, Runkang Qu, Yanan Zong, Chao Qin, Litao Liu, Xiaoyi Gao, Honglei Sun, Yipeng Sun, Kin-Chow Chang, Rui Zhang, Jinhua Liu, Juan Pu

**Affiliations:** 1 Key Laboratory for Prevention and Control of Avian Influenza and Other Major Poultry Diseases, Ministry of Agriculture and Rural Affairs, College of Veterinary Medicine, China Agricultural University, Beijing, China; 2 School of Veterinary Medicine and Science, University of Nottingham, Sutton Bonington Campus, Loughborough, United Kingdom; Emory University School of Medicine, UNITED STATES

## Abstract

Avian influenza virus (AIV) can evolve multiple strategies to combat host antiviral defenses and establish efficient infectivity in mammals, including humans. H9N2 AIV and its reassortants (such as H5N6 and H7N9 viruses) pose an increasing threat to human health; however, the mechanisms involved in their increased virulence remain poorly understood. We previously reported that the M1 mutation T37A has become predominant among chicken H9N2 isolates in China. Here, we report that, since 2010, this mutation has also been found in the majority of human isolates of H9N2 AIV and its emerging reassortants. The T37A mutation of M1 protein enhances the replication of H9N2 AIVs in mice and in human cells. Interestingly, having A37 instead of T37 increases the M1 protein stability and resistance to proteasomal degradation. Moreover, T37 of the H9N2 M1 protein is phosphorylated by protein kinase G (PKG), and this phosphorylation induces the rapid degradation of M1 and reduces viral replication. Similar effects are also observed in the novel H5N6 virus. Additionally, ubiquitination at K187 contributes to M1-37T degradation and decreased replication of the virus harboring T37 in the M1 protein. The prevailing AIVs thereby evolve a phospho-resistant mutation in the M1 protein to avoid viral protein degradation by host factors, which is advantageous in terms of replication in mammalian hosts.

## Author summary

H9N2 avian influenza virus (AIV) and its reassortants (such as H5N6 and H7N9 viruses) pose an increasing threat to human health, but the mechanisms involved in their increased virulence remain poorly understood. Notably, the role of viral M1 protein in increasing the mammalian infection of AIV has been rarely reported. Here, we demonstrate that a phospho-resistant T37A mutation, encoded by the M1 protein of recently prevalent chicken H9N2 virus, increases M1 protein stability and viral replication in mammalian cells. The T37, but not the A37, in H9N2 M1 protein can be phosphorylated by protein kinase G (PKG). Through the T37A mutation, viral M1 protein evades phosphorylation-mediated proteasomal degradation, resulting in increased avian H9N2 virus replication in mice and in human cells. Similar effects were also observed for the novel H5N6 virus. This study provides insight into a novel strategy by which AIV evades mammalian host defenses. It is necessary to pay close attention to the epidemiological and public health implications of AIVs carrying this mutant M1 protein.

## Introduction

H9N2 avian influenza viruses (AIVs) are prevalent worldwide among wild and domestic avian species, and pose a global threat to the poultry industry [1,2]. Recent research revealed that H9N2 AIVs could preferentially bind to the human-type sialic acid receptor and transmit efficiently between ferrets via respiratory droplets [3]. Notably, avian H9N2 virus caused 38 laboratory-confirmed human cases in China in 2020 and 2021. A total of 74 cases of infection with avian H9N2 virus were reported between 2013 and 2021, whereas only 10 cases were diagnosed from 1999 to 2012 (https://www.who.int/teams/global-influenza-programme/avian-influenza/monthly-risk-assessment-summary). Furthermore, H9N2 AIVs continue to reassort with other subtype viruses, resulting in the emergence of several novel subtypes (e.g., H5N6, H7N9, and H10N8 viruses) that cause severe disease in infected humans [4–6]. This evidence indicates that the prevailing H9N2 virus and its reassortants undergo more efficient inter-species transmission compared with previously dominant AIVs and, therefore, pose a growing threat to human health.

Phosphorylation is a reversible post-translational protein modification, usually targeting a serine, threonine, or tyrosine residue, that regulates protein functions, including enzyme activity, signaling transduction, protein stability, and subcellular localization [7,8]. Protein phosphorylation followed by ubiquitination and proteasomal degradation is a mechanism commonly used by cells to degrade proteins in a controllable manner [7,9]. Protein phosphorylation can either induce the exposure of a degradation sequence or provide a recognition site for the coupled E3 ubiquitin ligase complex, resulting in the degradation of proteins and a change of function [10,11]. Phosphorylated viral proteins of influenza viruses typically interact with host factors and thereby affect viral replication [12–17]. For example, a loss of phosphorylation at tyrosine 132 on the M1 protein of A/WSN/33 (WSN) virus inhibits viral replication by disrupting the importin-α1-mediated nuclear import of M1 [12]. Phosphorylation of threonine 49 on NS1 protein impairs the binding of NS1 to double-stranded RNA and TRIM25, as well as complex formation with RIG-I, resulting in neutralization of the ability of NS1 to inhibit a host type I interferon response [13]. However, all of these effects of phosphorylation are demonstrated in model viruses and with artificial viral mutations; very few studies have reported the phosphorylation-dependent degradation of influenza virus proteins.

The most abundant and conservative viral protein, M1 protein, which is encoded by the M gene, plays multiple roles in the influenza virus life cycle [18–21]. However, little is known regarding the role of viral M1 protein in increasing the mammalian infection of AIV. Our previous study discovered that threonine 37 (T37) of the M1 protein of early H9N2 AIVs was gradually replaced by alanine, and alanine 37 (A37) is highly representative and conserved in the M1 protein of the prevailing chicken H9N2 virus [22]. Our preliminary analysis revealed that A37 of M1 protein was also present in a high percentage in the H9N2 viruses isolated from humans, suggesting that this mutation may be delivered from chicken isolates to human isolates and thus related to the observed increase in human infections with avian H9N2 virus in recent years. A previous mass spectrometry-based study speculated that T37 on the M1 protein of influenza A virus may be a putative phosphorylation site [23]. We hypothesize that the evolutionary change on the M1 protein of H9N2 AIVs from threonine to alanine at position 37 results in the natural loss of phosphorylation and a functional gain in the replication ability of H9N2 viruses in mammalian hosts.

We demonstrate here that T37 in the M1 protein of early avian H9N2 virus is phosphorylated by protein kinase G (PKG), but the A37 substitution encoded by the recently prevalent chicken H9N2 virus leads to a loss of phosphorylation. M1 protein evaded phosphorylation-mediated proteasomal degradation through this mutation, resulting in the enhanced replication of avian H9N2 influenza virus in mammals. The novel H5N6 virus with A37 in its M1 protein also showed increased protein stability and viral replication. These findings provide a possible explanation for the increasing number of human cases of infection with H9N2 virus and its reassortants in recent years, and reveal a novel strategy by which AIVs can evade mammalian host defenses.

## Results

### M1 protein harboring the T37A mutation has become predominant among the prevailing H9N2 virus and its emerging reassortants isolated from humans

Most human cases of infection with avian H9N2 virus are exposed to the virus via contact with infected poultry or contaminated environments. Our previous study discovered that among the prevailing chicken H9N2 viruses in China, M1 protein with an amino acid residue substitution from T to A at position 37 has become dominant. This mutation confers increased virus replication ability in chicken embryo fibroblasts (CEFs) and chickens. [22]. To determine whether this amino acid change has been delivered from chicken isolates to human isolates, we analyzed the available M1 sequences of avian H9N2 viruses isolated from humans. Sequence searches revealed that 75.00% of the M1 proteins of H9N2 viruses isolated from humans during the period from 1999–2009 carried the T37. However, for H9N2 viruses isolated after 2010, this percentage decreased sharply, and all human isolates of the H9N2 virus harbored A37 in the M1 protein (Table 1); this occurrence coincided with the increase in human H9N2 infection cases and countrywide H9N2 virus outbreaks in chickens. As the prevailing H9N2 viruses provide up to six internal genes to the novel H5N6, H7N9, and H10N8 viruses, we investigated the presence of M1 proteins with this mutation among human isolates. As shown in Table 1, H5N6, H7N9, and H10N8 strains isolated from humans showed low frequencies of the T37 residue, only 6.25%, 0.36%, and 0%, respectively; in contrast, M1 proteins harboring A37 were present at higher frequencies, 93.75%, 99.20%, and 100%, respectively. Because H9N2 and its reassortant viruses have continued to cause infections in humans in recent years, these findings suggest that the M1 protein change from T37 to A37 may be involved in the increased infection of avian H9N2 virus in humans.

**Table 1:** Prevalence of amino acid residues at position 37 of the M1 protein in H9N2 influenza virus and its reassortants isolated from humans.

Subtypes	Epidemic time	Prevalence in human viruses (%)^a^		
		M1 protein with T37	M1 protein with A37	Other
H9N2	1999–2009	75.00	25.00	0
	2010–2021	0	100.00	0
H5N6	2014–2021	6.25	93.75	0
H7N9	2013–2021	0.36	99.20	0.44
H10N8	2013–2021	0	100.00	0

^a^ The total numbers of avian H9N2 (1999–2009), H9N2 (2010–2021), H5N6, H7N9, and H10N8 viruses isolated from humans were 8, 36, 32, 1368, and 3, respectively. Available M1 protein sequences from emergence to date (year 2021) were downloaded from the GISAID and NCBI databases. Viruses repeated in both databases were merged before analysis.

### M1 protein with T37A mutation confers increased pathogenicity and replication of H9N2 virus in mice

**Fig 1:**
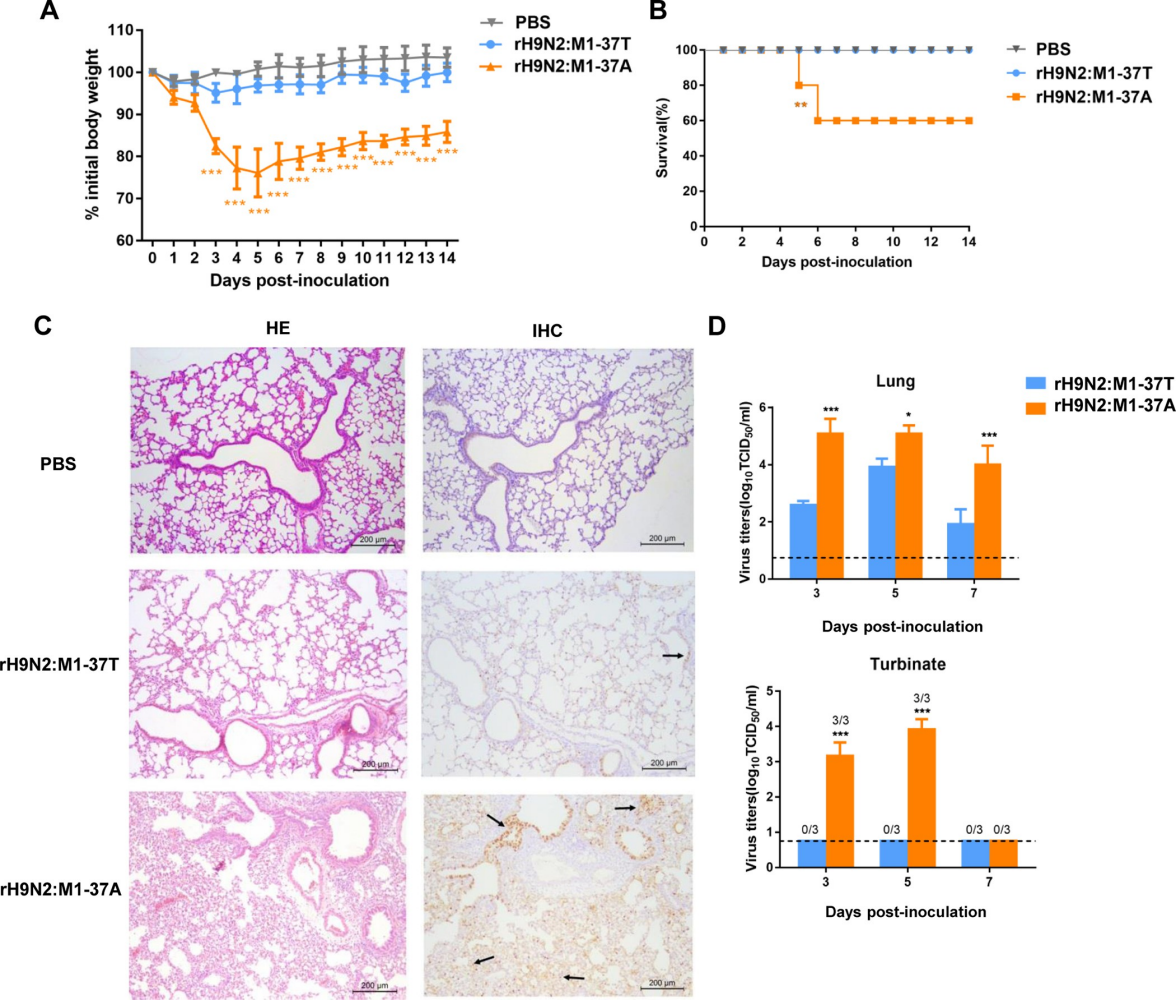
M1 protein with T37A mutation confers increased pathogenicity and replication of H9N2 virus in mice. Six-week-old female BALB/c mice were individually inoculated with 10^6^ TCID_50_ of rH9N2:M1-37T or rH9N2:M1-37A viruses, or were mock infected with PBS via the intranasal route. (A) Body weight changes in mice over a 14-day period (n = 5 per group in one independent experiment). Each data point represents the mean ± standard deviation (SD) and is representative of three independent experiments. Statistical significance was based on two-way ANOVA (***P<0.001). (B) Survival rate of mice. Mice that lost more than 30% of their baseline weight were euthanized. Data were pooled from three independent experiments with n = 5 mice per group. Survival curves were compared using log-rank (Mantel–Cox) analysis. **P<0.01. (C) Representative hematoxylin and eosin (H&E)-stained and immunohistochemistry (IHC) examination of lung sections from three mice infected with each indicated virus at 3 days post-infection (dpi). Arrows indicate viral NP localizations. Scale bar, 200 μm. (D) Virus titers recovered from mouse lungs and turbinates, harvested from three mice per group at 3, 5, and 7 dpi. Each data point represents the mean ± SD and is representative of three independent experiments. Dashed lines indicate the lower limit of detection. Statistical significance was based on two-way ANOVA (*P<0.05; **P<0.01; ***P<0.001).

To investigate the influence of the spontaneous M1 T37A mutation of avian H9N2 virus on viral pathogenicity and replication in mammals, we generated virus rH9N2:M1-37T from a wild-type H9N2 isolate (A/chicken/Shandong/Lx1023/2007, abbreviated as lx1023) that contains T37 in the M1 protein, and rH9N2:M1-37A virus, which differs from rH9N2:M1-37T by a single alanine substitution at position 37 of the M1 protein. Each virus was intranasally inoculated at 10^6^ TCID_50_ into BALB/c mice, which were then monitored over 14 days for weight loss and survival. All mice infected with the rH9N2:M1-37T virus survived and showed no adverse change in body weight. In contrast, mice infected with the rH9N2:M1-37A virus showed significant weight loss (P<0.001) (Fig 1A), and two of these mice died by 6 days post-infection (dpi) (Fig 1B). Infected lung tissues were collected at 3 dpi for histopathological analysis. Lungs from rH9N2:M1-37T-infected mice appeared almost identical to those from uninfected controls. However, the rH9N2:M1-37A virus caused severe interstitial pneumonia and bronchopneumonia, which was characterized by alveolar consolidation and extensive inflammatory cell infiltration. As expected, viruses were extensively detected by viral nucleoprotein (NP)-specific antibodies in the lungs of rH9N2:M1-37A-infected mice, but were barely detectable in the lungs of the rH9N2:M1-37T group mice (Fig 1C).

Viral titers in nasal turbinates and the lungs of infected mice were determined at 3, 5, and 7 dpi. Consistent with the pathogenicity results, the virus carrying T37 produced significantly lower titers in the lungs compared with the rH9N2:M1-37A virus at 3, 5, and 7 dpi (up to 300-fold lower, P<0.001) (Fig 1D). More significant differences in virus output were observed from the nasal turbinates. The rH9N2:M1-37A virus exhibited efficient replication, whereas the progeny virus of rH9N2:M1-37T was below the detection threshold in the nasal turbinates of mice throughout the experimental period (Fig 1D).

Collectively, avian H9N2 virus harboring the T37A mutation in the M1 protein showed elevated viral pathogenicity and replication in mice. This finding correlates with the ongoing increase in the number of H9N2 virus clinical infections in humans that is occurring as viruses with this mutation are becoming more prevalent.

### The T37A substitution in M1 protein is associated with increased production of H9N2 virus in human A549 cells

**Fig 2:**
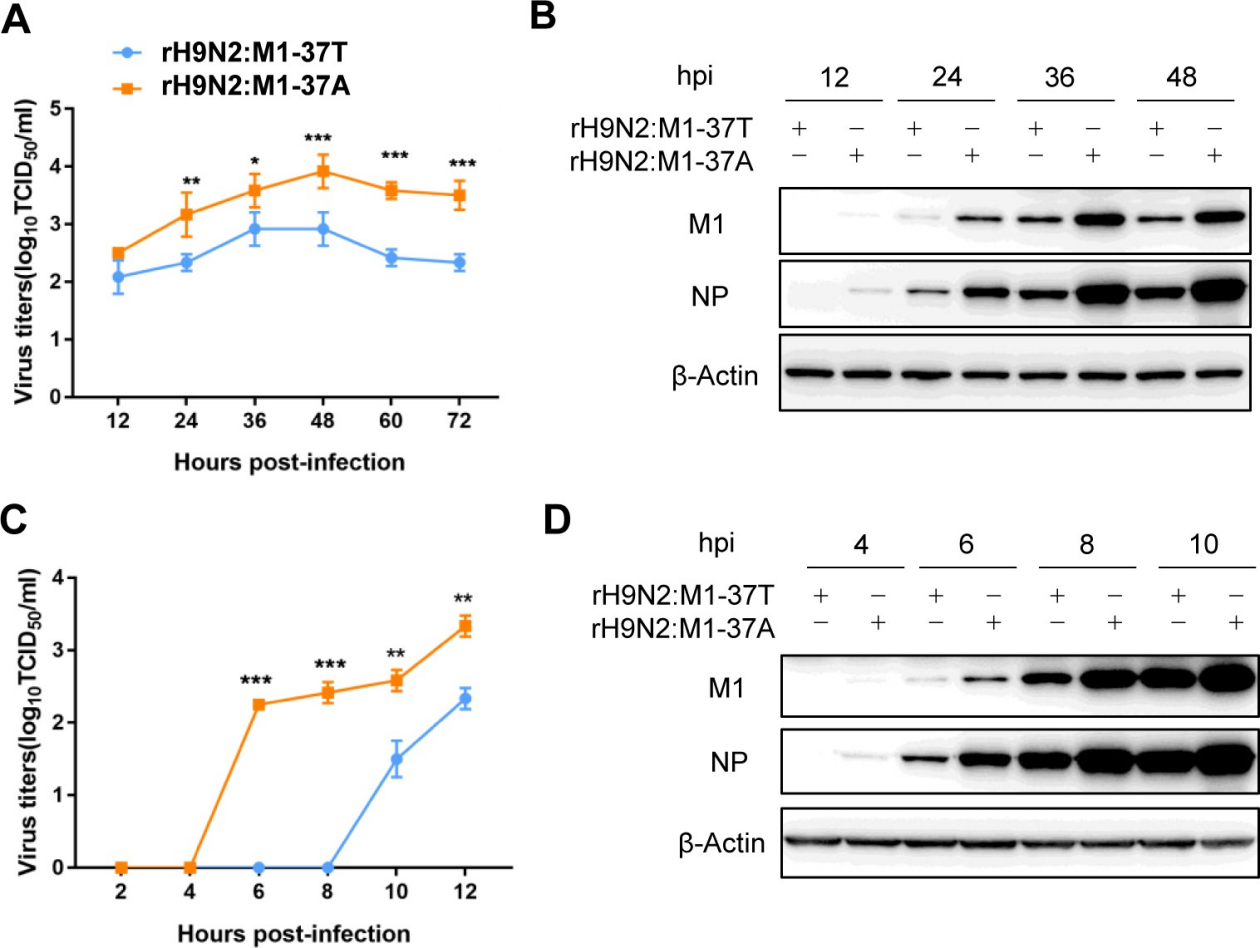
The T37A substitution in M1 protein is associated with increased production of H9N2 virus in human A549 cells. A549 cells were infected with the rH9N2:M1-37T or rH9N2:M1-37A virus at an multiplicity of infection (MOI) of 0.2 (A, B) or 1 (C, D). (A, C) Virus output of rH9N2:M1-37T and rH9N2:M1-37A viruses from infected A549 cells. Multistep growth curves of H9N2 viruses (A) and single replication cycle of H9N2 viruses (C). Virus titers were determined from supernatants collected at the indicated time points. Data are presented as the mean ± SD of three independent experiments. Statistical significance was based on two-way ANOVA (*P<0.05; **P<0.01; ***P<0.001). (B, D) Viral M1 and NP production in A549 cells. A549 cells were infected with the rH9N2:M1-37T or rH9N2:M1-37A virus and harvested at the indicated time points; western blotting (WB) was then performed on the cell lysates. The panels show viral protein expression at 12, 24, 36, and 48 hours post-infection (hpi) (B), or at 4, 6, 8, and 10 hpi (D). β-Actin was used as a loading control. The WB results are representative of three independent experiments.

To further examine the role of the M1 T37A mutation in virus replication, we infected human A549 cells with rH9N2:M1-37T or rH9N2:M1-37A viruses over 72 h and performed a multistep replication kinetics analysis. rH9N2:M1-37A grew to significantly higher titers (over 10-fold higher, P<0.001) compared with rH9N2:M1-37T virus from 24 to 72 h post-infection (hpi) (Fig 2A). Viral M1 and NP protein levels were correspondingly higher in the rH9N2:M1-37A group (Fig 2B). We further conducted a single-replication-cycle kinetics analysis over 12 h of infection. Progeny rH9N2:M1-37A virus was detected at 6 hpi, approximately 4 h ahead of rH9N2:M1-37T progeny detection, and showed significantly higher levels (P<0.001) than those of rH9N2:M1-37T virus at each time point (Fig 2C). Similarly, the M1 and NP proteins of rH9N2:M1-37A were first detected at 4 hpi, around 2 h prior to the detection of the viral proteins of rH9N2:M1-37T virus (Fig 2D). These results demonstrated that the M1 T37A mutation confers more rapid and higher production of progeny avian H9N2 virus in human A549 cells.

### The stability of H9N2 M1 protein is increased by the replacement of T37 with A37

Abundant M1 protein is necessary for the efficient replication of influenza viruses. We therefore examined whether the A37 mutation could affect protein levels by constructing the corresponding M1 expression plasmids from lx1023 virus, designated M1-37T and M1-37A.

**Fig 3:**
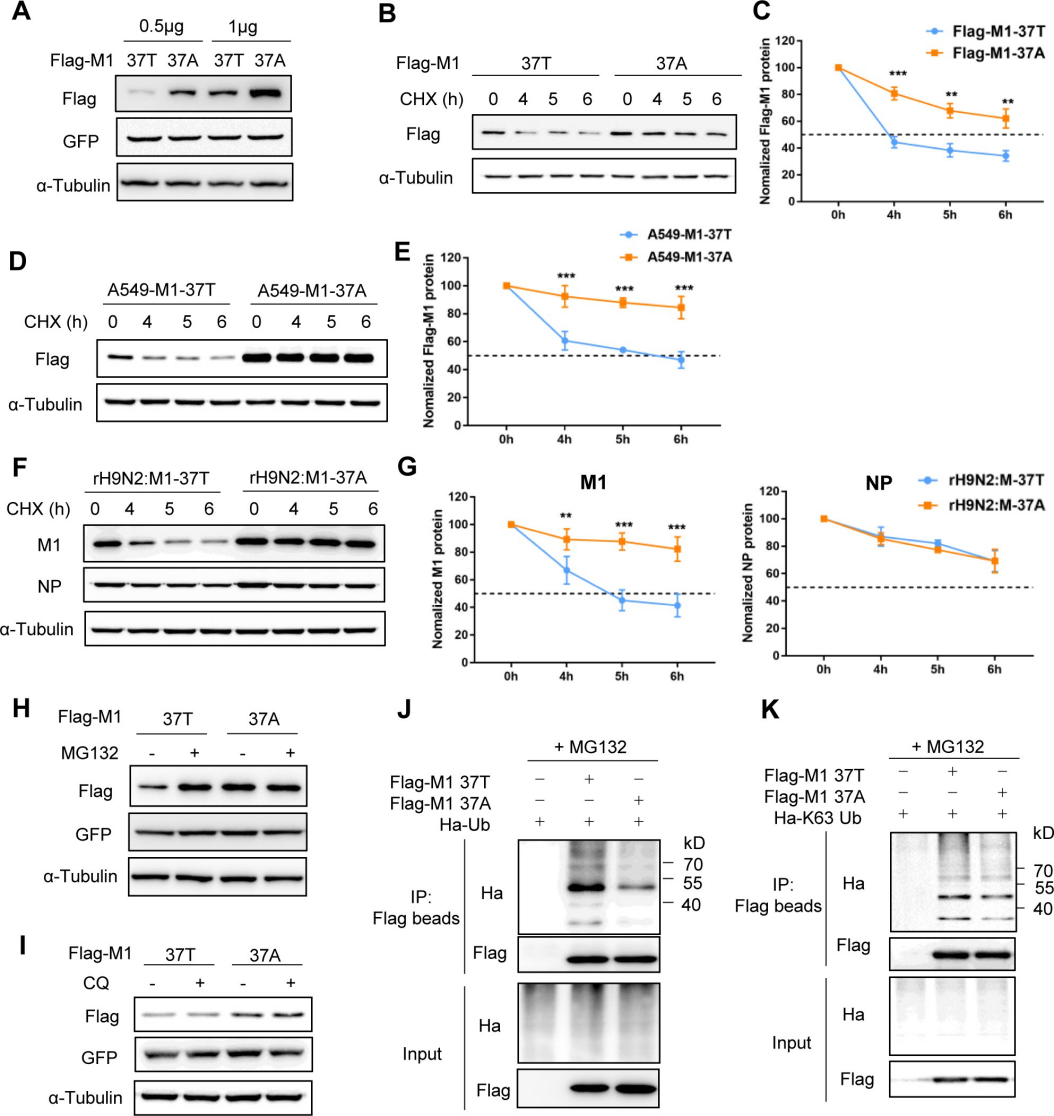
The stability of H9N2 M1 protein is increased by the replacement of T37 with A37. (A) The protein abundance levels of M1-37T and M1-37A at different transfection doses. HEK293T cells were co-transfected with the indicated dose of Flag-tagged M1 (37T or 37A) and pEGFPC1 plasmids. Western blotting (WB) was performed to analyze the expression levels of Flag-M1 and GFP. GFP was used as a transfection control and α-Tubulin was used as a loading control. (B–G) Protein degradation assay of M1 protein. (B, C) A549 cells were transfected with Flag-tagged M1 (37T or 37A) expression plasmids for 24 h, followed by CHX (50 μg/mL) treatment over the indicated time course. (D, E) Protein degradation assay of M1 protein in the stable M1-overexpressing A549 cell lines (A549-M1-37T and A549-M1-37A). (F, G) A549 cells were infected with rH9N2:M1-37T or rH9N2:M1-37A for 24 h and then treated with CHX. NP protein was used as a viral protein control, and α-Tubulin was used as a loading control. Densitometry analysis of the data presented in Fig B, D, and F are displayed as respective graphs (Fig C, E, and G), and the data represent the mean ± SD pooled from three independent experiments. Statistical significance was based on two-way ANOVA (**P<0.01; ***P<0.001). (H, I) WB analysis to detect the expression levels of M1 in A549 cells transfected with Flag-tagged M1 (37T or 37A) plasmids for 24 h, followed by MG132 (20 μM, H) or CQ (150 μM, I) treatment for 6 h. (J) Ubiquitination analysis of M1. HEK293T cells were co-transfected with Ha-tagged ubiquitin (Ha-Ub) plasmids and Flag-tagged M1 or empty vector plasmid; 24 h later, the cells were treated with MG132 (20 μM) for 6 h. Ubiquitinated proteins were then analyzed by WB. (K) HEK293T cells were co-transfected with Flag-tagged M1 (37T or 37A) or empty vector plasmid, and plasmid encoding a version of ubiquitin capable of binding the substrate only through K63; 24 h later, the cells were treated with MG132 for 6 h. Ubiquitinated proteins were then analyzed by WB. All WB results are representative of three independent experiments.

First, we transfected HEK293T cells with plasmids expressing M1-37T or M1-37A in different doses. Immunoblotting analysis revealed the higher expression of M1-37A than of M1-37T at each transfection dose (Figs 3A and S1A). To further determine whether this difference was caused by a difference in protein stability, we determined the protein half-life of M1-37T and M1-37A by treating transfected cells with the protein synthesis inhibitor cycloheximide (CHX) over a time course, followed by western blot analysis (Fig 3B). The half-life of M1-37A (>6 h) was clearly longer than that of M1-37T (~4 h) (Fig 3B and 3C), while the mRNA level of M1-37A was similar to that of M1-37T (S1B Fig). To eliminate the unstable factors of transient transfection, we further transduced A549 cells separately with lentivirus carrying H9N2 M1-37T or M1-37A to establish two stable cell lines. As expected, a similar result was found in the stable cell lines (Fig 3D and 3E). Consistent with this, the M1 half-life during rH9N2:M1-37A virus infection in A549 cells was also longer than that during rH9N2:M1-37T virus infection, but no noticeable difference in half-life was observed between the two NP proteins (Fig 3F and 3G), indicating the greater stability of M1-37A over M1-37T protein during viral infection.

To determine the pathway of M1-37T degradation, we treated M1-37T- and M1-37A- transfected cells with the proteasome inhibitor MG132 or with one of the lysosome inhibitors chloroquine (CQ) and NH_4_Cl in parallel experiments. We found that MG132 treatment significantly rescued the M1-37T protein level but had no apparent effect on the M1-37A protein level, and the protein level of MG132-treated M1-37T was similar to that of M1-37A, indicating that M1-37T was targeted by the proteasome-degradation pathway (Figs 3H and S1C). In contrast, treatment with lysosome inhibitors CQ or NH_4_Cl had little effect on the expression of M1-37T protein (Figs 3I and S1D–S1F).

To further determine whether ubiquitination mediates the proteasomal degradation of M1-37T, we performed a ubiquitination assay. The ubiquitination level of M1-37T was much higher than that of M1-37A after inhibiting proteasomal function with MG132 (Fig 3J). Ubiquitin can bind to its substrates through different types of homogeneous polyubiquitin chains. K48- and K63-linked polyubiquitin chains are the two most abundant polyubiquitin chain types [24]. We observed that the ubiquitination of M1-37T occurred through K63 linkage and not through K48 linkage, demonstrating that the K63-linked ubiquitination of M1 protein promotes its proteasomal degradation (Figs 3K and S1G). These findings suggest that the replacement of T37 with A37 increases the stability of H9N2 M1 protein and reduces M1 degradation by the ubiquitin-proteasome pathway.

### T37 of the H9N2 M1 protein is phosphorylated by PKG

Protein phosphorylation can lead to proteasomal degradation, which is a commonly used pathway in the cell life cycle [7,25]. T37 in M1 protein was predicated as a potential phosphorylated residue and PKG is the potential kinase for this site in influenza A virus [23]. Alanine is usually introduced into a threonine/serine site to mimic phosphorylation-resistant residues [12,26]. Thus, H9N2 viruses might naturally lose the potential phosphorylation site T37 in the M1 protein.

**Fig 4:**
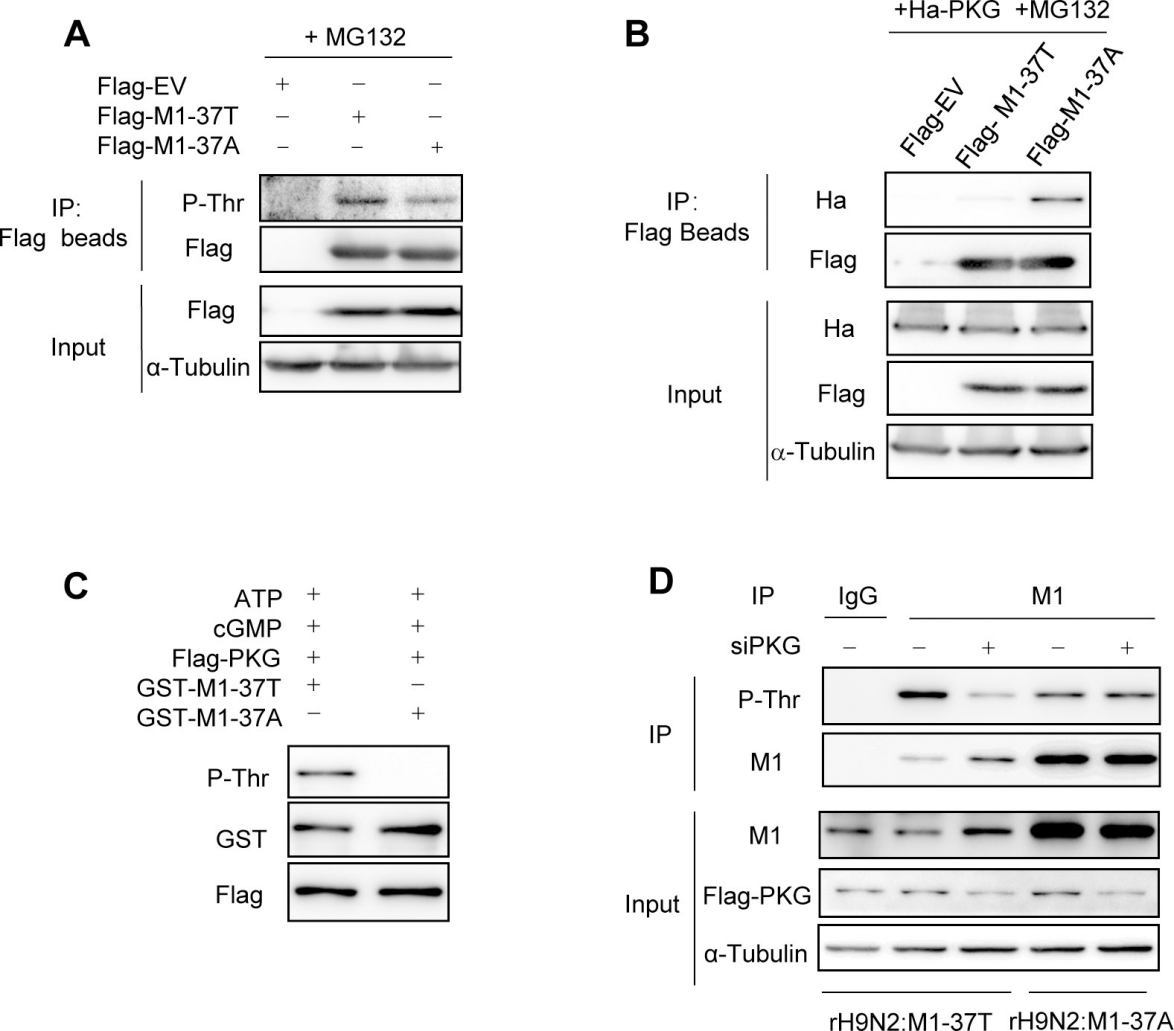
T37 of the H9N2 M1 protein is phosphorylated by PKG. (A) Immunoblotting analysis (IB) analysis of the threonine phosphorylation levels of M1 protein. HEK293T cells transfected with Flag-tagged M1 (37T or 37A) expression plasmids or empty vector (Flag-EV) plasmid for 24 h, followed by MG132 (20 μM) treatment for 6 h, were immunoprecipitated with Flag beads. Threonine phosphorylation levels of M1 were detected by an anti-phosphothreonine antibody. (B) Interaction between M1 and PKG. IB analysis of immunoprecipitates of HEK293T cells co-transfected with Flag-tagged M1 (37T or 37A) and Ha-tagged PKG expression plasmids. (C) *In vitro* kinase assay. Bacterially-purified GST-M1 (37T or 37A) and purified Flag-PKG from HEK293T cells were incubated in the presence of ATP and cGMP *in vitro*. IB was performed to analyze the threonine phosphorylation levels of M1, GST-M1, and Flag-PKG. (D) IB analysis of the threonine phosphorylation levels of M1 in the immunoprecipitates of control or PKG-silenced A549^Flag-PKG^ cells infected with rH9N2:M1-37T or rH9N2:M1-37A for 24 h. α-Tubulin was used as a loading control. The western blotting results are representative of three independent experiments.

To verify that the stability of M1 protein is related to phosphorylation-dependent degradation, we first needed to confirm that M1 T37 is a phosphorylation site. We transfected HEK293T cells with plasmids expressing Flag-tagged M1-37T and M1-37A and then analyzed the phosphothreonine level using an anti-phosphothreonine antibody after pulling down M1 proteins. The results showed that the conversion from T37 to A37 greatly reduced the level of M1 threonine phosphorylation (Figs 4A and S2A), indicating that T37 is a phosphothreonine site on M1.

To evaluate whether PKG is the kinase responsible for T37 phosphorylation, we first investigated the interaction between M1 and PKG. PKG was easily detected in the precipitates of M1-37A but was less readily detected in those of M1-37T (Fig 4B), suggesting that the binding of PKG with its substrate, M1-37T, was transient, as are many kinase–substrate interactions [27–29]. We then performed an *in vitro* kinase assay, which is recognized as the most direct approach for verifying the substrate of a kinase [30,31]. Bacterially expressed and purified GST-M1 proteins and purified Flag-tagged PKG protein from HEK293T cells were co-incubated in the presence of ATP and cGMP *in vitro*. The results showed that PKG phosphorylated M1-37T but not M1-37A, which confirmed that the T37 of M1 protein is a phosphorylation site catalyzed by PKG (Fig 4C).

Next, we determined whether PKG affects the phosphorylation status of M1 protein during viral infection. We constructed an A549 cell line that expresses Flag-PKG via introducing *Flag* to the endogenous *PKG* locus using CRISPR-Cas9 technique described previously [32]. We transfected the resulting A549^Flag-PKG^ cells with control or PKG siRNAs, and then the transfected cells were infected rH9N2:M1-37T or rH9N2:M1-37A virus, respectively. After immunoprecipitation with anti-M1 antibody, the M1 threonine phosphorylation levels were evaluated. PKG knockdown dramatically reduced the M1 threonine phosphorylation levels of rH9N2:M1-37T virus but had little effect on those of rH9N2:M1-37A virus (Figs 4D and S2B). Moreover, the rH9N2:M1-37A virus exhibited lower basal M1 phosphothreonine levels, although it produced more total M1 protein compared with the rH9N2:M1-37T virus (Fig 4D). Collectively, T37 but not A37 of the M1 protein is phosphorylated by PKG.

### Loss of T37 phosphorylation protects M1 protein from PKG-directed degradation

**Fig 5:**
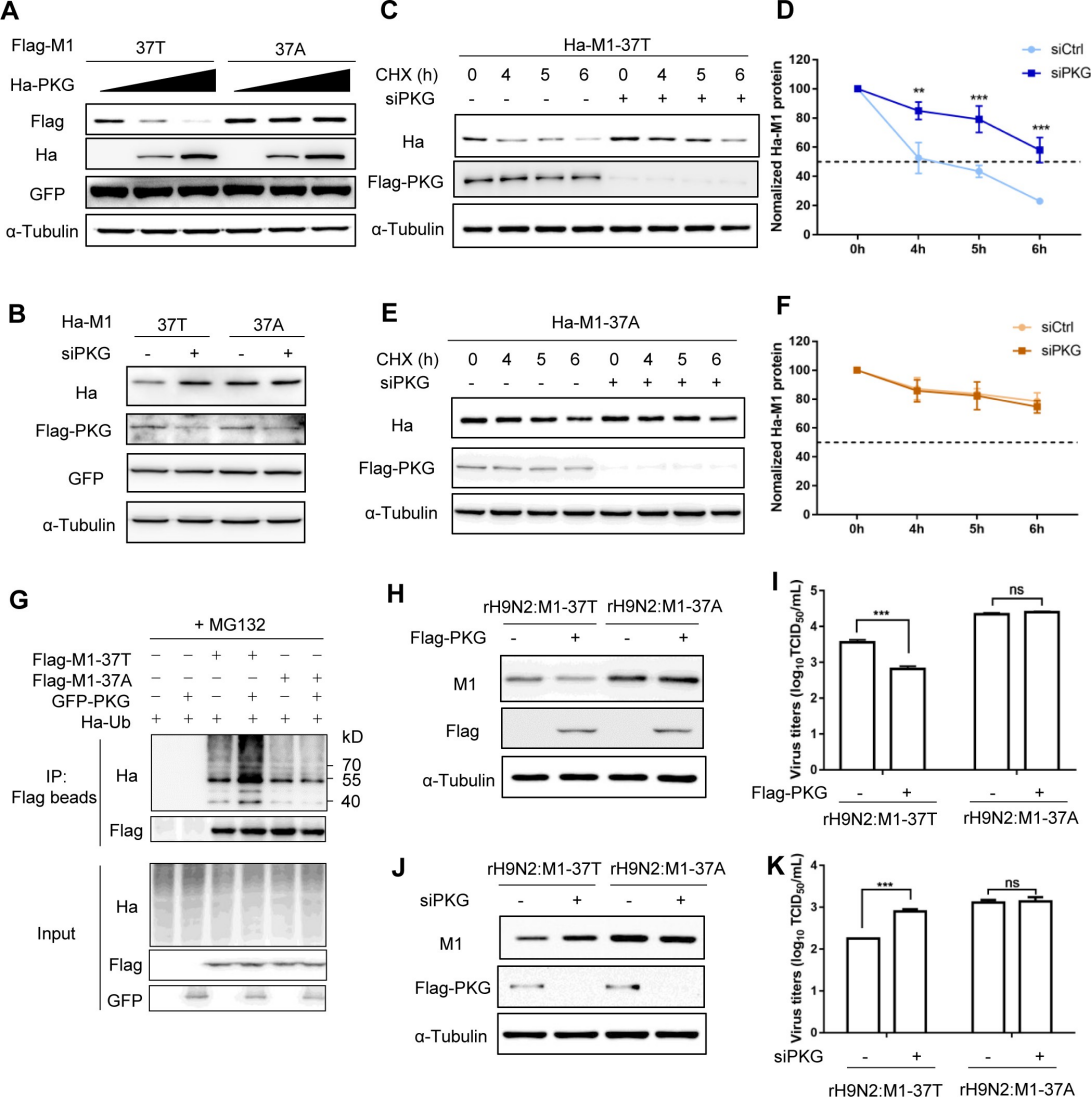
Loss of T37 phosphorylation protects M1 protein from degradation directed by PKG. (A) HEK293T cells were co-transfected with Flag-tagged M1 (37T or 37A), pEGFPC1, and an increasing dose of Ha-PKG expression plasmids. Western blotting (WB) was performed to analyze the expression levels of M1, GFP, and PKG. (B) WB analysis of the expression levels of Ha-M1, Flag-PKG, and GFP in control or PKG-silenced A549^Flag-PKG^ cells transfected with Ha-tagged M1 and GFP expression plasmids. GFP was used as a transfection control and α-Tubulin was used as a loading control. (C–F) WB analysis of the half-life of M1 protein in control or PKG-silenced A549^Flag-PKG^ cells transfected with M1 expression plasmids. Control or PKG-silenced A549^Flag-PKG^ cells were transfected with Ha-tagged M1-37T (C) or M1-37A (E) expression plasmids for 24 h, then treated with CHX (50 μg/mL) for the indicated time. WB was performed to analyze the expression levels of Ha-M1 and Flag-PKG. Data presented in Fig C and E were quantified as the ratio of Ha-M1 to α-Tubulin and were displayed in respective graphs (D, F). The data represent the mean ± SD pooled from three independent experiments. Statistical significance was based on two-way ANOVA (**P<0.01; ***P<0.001). (G) The effect of PKG on the ubiquitination of M1 proteins. Ha-Ub plasmids were co-transfected in HEK293T cells with GFP-PKG and Flag-M1 or empty plasmids for 24 h, followed by MG132 (20 μM) treatment for 6 h. Ubiquitinated proteins were then analyzed by WB. (H) WB analysis of M1 and PKG expression in A549 cells that had been transfected with Flag-PKG expression plasmids for 24 h, followed by rH9N2:M1-37T or rH9N2:M1-37A virus infection for 24 h. (I) Virus titers in supernatants, as described in (H), were analyzed to determine the TCID_50_. (J) WB analysis of M1 and Flag-PKG in A549^Flag-PKG^ cells transfected with control or PKG siRNAs for 24 h, followed by rH9N2:M1-37T or rH9N2:M1-37A infection for 24 h. (K) Virus titers in supernatants, as described in (J), were analyzed by TCID_50_. Error bars in (I) and (K) represent the SD from the mean for three independent experiments. Statistical significance was based on *t*-tests (***P<0.001). All WB data are representative of three independent experiments showing similar results.

We predicted that if the stability of M1 protein is related to phosphorylation-dependent degradation, PKG, the kinase for T37 in the M1 protein, would play a critical role. To determine whether PKG could degrade M1, we co-transfected HEK293T cells with either M1-37T or M1-37A plasmids and increasing amounts of PKG expression plasmids. Immunoblot analysis revealed that increasing amounts of PKG steadily reduced the levels of M1-37T protein; however, the expression of M1-37A showed no obvious change (Fig 5A). When endogenous PKG in A549^Flag-PKG^ cells was knocked down by using siRNAs, more accumulated M1-37T proteins were observed, but the M1-37A protein levels were not affected (Fig 5B). We further analyzed the effect of PKG on the half-life of M1. As shown in Fig 5C–5F, PKG depletion increased the expression levels and half-life of M1-37T protein but had no apparent effect on the stability of M1-37A protein. Consistent with this, PKG overexpression enhanced the ubiquitination of M1-37T but had little impact on M1-37A ubiquitination (Fig 5G). These results demonstrated that M1-37A is resistant to degradation directed by PKG.

The increased stability of M1 protein may contribute to increased viral replication. We found that PKG overexpression significantly reduced the M1 expression and number of progeny viruses upon rH9N2:M1-37T infection but had little effect on these properties in rH9N2:M1-37A-infected cells (Figs 5H, S3A and 5I). Accordingly, in PKG-depleted A549^Flag-PKG^ cells, rH9N2:M1-37T virus produced more M1 protein and progeny virus compared with the control-treated cells (Figs 5J, S3B and 5K). Again, M1 protein expression and progeny virus released from rH9N2:M1-37A-infected cells remained largely unchanged regardless of PKG knockdown (Figs 5J, S3B and 5K). In addition, PKG activity was significantly increased in response to both viral infections, despite its protein levels remaining unchanged (S3C and S3D Fig). In summary, this spontaneous M1 mutation, via the loss of phosphorylation, protects M1 protein from degradation directed by PKG and thereby promotes viral replication.

To further confirm the effect of the T37A mutation on M1 protein stability in the H9N2 virus, we generated another two M1 plasmids derived from a chicken strain (A/Chicken/Beijing/3/1999, abbreviated as BJ/3/99), designated BJ-M1-37T and BJ-M1-37A. Similarly, BJ-M1-37A showed a higher protein level (S4A and S4B Fig) and a longer half-life in both cases of transfection (S4C and S4D Fig) and of viral infection (S4E and S4F Fig) compared with those of BJ-M1-37T. Treatment with MG132 (S4G and S4H Fig) but not with CQ (S4I and S4J Fig) inhibited the degradation of BJ-M1-37T protein while having little effect on that of BJ-M1-37A protein. The conversion from T37 to A37 also significantly reduced the levels of M1 ubiquitination and M1 threonine phosphorylation (S4K and S5A Figs). Moreover, PKG depletion in A549^Flag-PKG^ cells raised the half-life of BJ-M1-37T from 4 h to 6 h (S5B and S5C Fig) but had no apparent effect on that of BJ-M1-37A (S5D and S5E Fig). Additionally, we rescued another group of recombinant H9N2 viruses derived from BJ/3/99 virus, rBJ/3/99:M1-37T and rBJ/3/99:M1-37A, which carried T37 and a single A37 substitution on M1 protein, respectively. rBJ/3/99:M1-37A grew to higher titers from 36 to 60 hpi compared with rBJ/3/99:M1-37T virus in A549 cells (S5F Fig). Mice infected with the rBJ/3/99:M1-37A virus showed significantly more weight loss and higher viral titers in the lungs and turbinates compared with mice infected with the rBJ/3/99:M1-37T virus (S5G–S5I Fig). These results further indicated that the T37A mutation can increase M1 protein stability and facilitate the viral replication of avian H9N2 virus in human cells and in mice.

### The T37A substitution increases both H5N6 M1 protein stability and H5N6 viral replication in mammals

**Fig 6:**
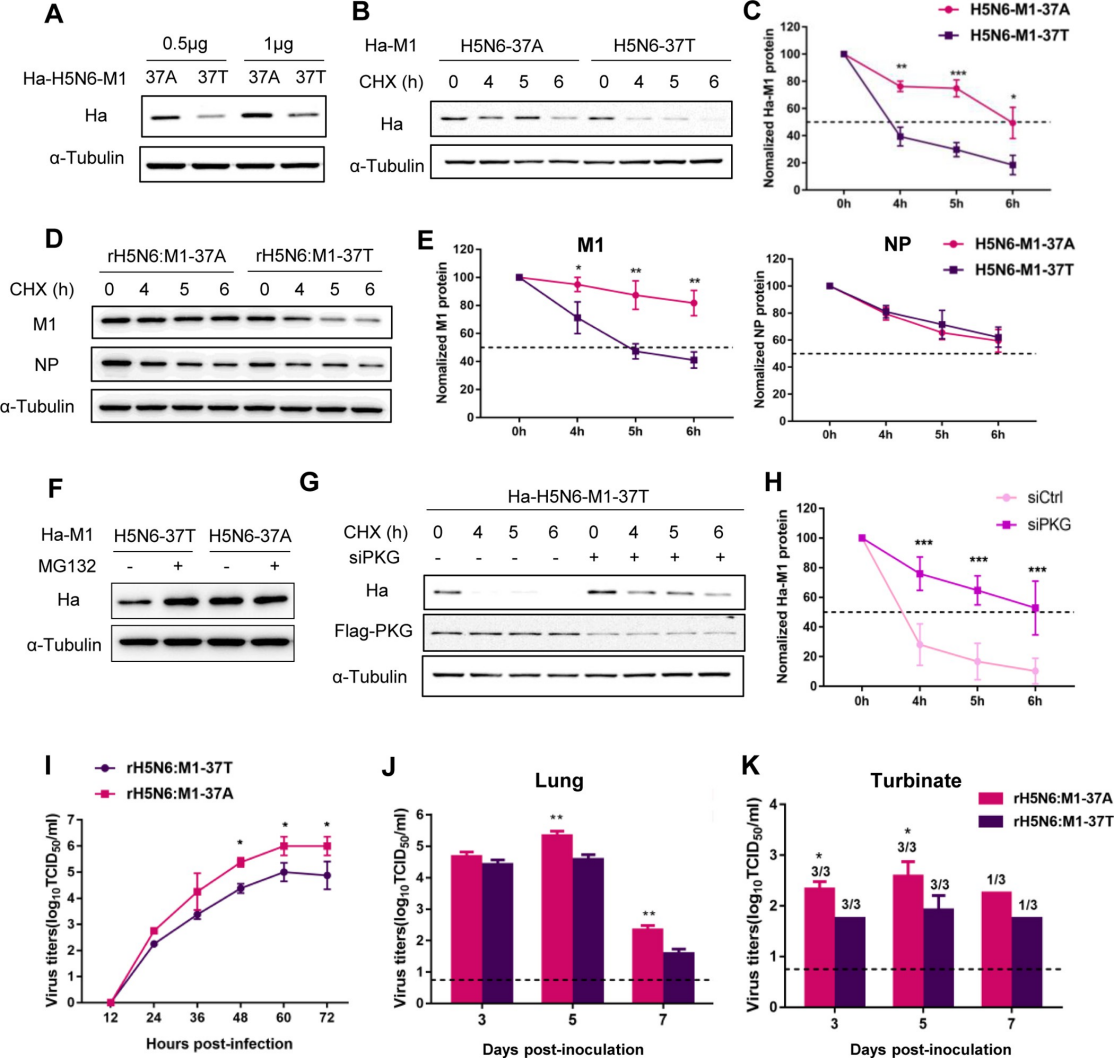
The T37A substitution increases both H5N6 M1 protein stability and H5N6 viral replication in mammals. (A) The protein expression levels of H5N6 M1 proteins at different transfection doses. HEK293T cells were transfected with the indicated dose of Ha-tagged H5N6 M1 (H5N6-M1-37A or H5N6-M1-37T) expression plasmids. Western blotting (WB) was performed to analyze the expression levels of Ha-M1. α-Tubulin was used as a loading control. (B–E) Protein degradation assay of H5N6 M1 protein. A549 cells were transfected with Ha-tagged H5N6 M1 (37A or 37T) expression plasmids (B) or were infected with rH5N6:M1-37A or rH5N6:M1-37T (D) for 24 h, followed by CHX (50 μg/mL) treatment over indicated time course. α-Tubulin was used as a loading control. Densitometry analysis of the data presented in (B, D) are displayed in respective graphs (C, E), and the data represent the mean ± SD pooled from three independent experiments. Statistical significance was based on two-way ANOVA (**P<0.01; ***P<0.001). (F) WB analysis of the expression levels of H5N6-M1 in A549 cells transfected with Ha-tagged H5N6 M1 plasmids followed by MG132 (20 μM) treatment for 6 h. (G) WB analysis of the half-life of H5N6 M1 protein in control or PKG-silenced A549^Flag-PKG^ cells. Control or PKG-silenced A549^Flag-PKG^ cells were transfected with Ha-tagged H5N6-M1-37T plasmid for 24 h, then treated with CHX for the indicated time. WB was performed to analyze the expression levels of Ha-M1 and Flag-PKG. Data were quantified as the ratio of Ha-M1 to α-Tubulin and are displayed in graph (H). The data represent the mean ± SD pooled from three independent experiments. Statistical significance was based on two-way ANOVA (**P<0.01; ***P<0.001). All WB data are representative of three independent experiments showing similar results. (I) Virus output of rH5N6:M1-37T and rH5N6:M1-37A viruses from infected A549 cells. A549 cells were infected at a multiplicity of infection (MOI) of 0.001. Virus titers were determined from supernatants collected at the indicated time points. Error bars represent the SD from three independent experiments. Statistical significance was based on two-way ANOVA (*P<0.05). (J, K) Virus titers recovered from mouse lungs (J) and turbinates (K). Lungs and turbinates were harvested from three mice per group at 3, 5, and 7 dpi for virus titration. Each data point represents the mean ± SD and is representative of three independent experiments. Dashed lines indicate the lower limit of detection. Statistical significance was based on two-way ANOVA (*P<0.05; **P<0.01).

To assess whether the A37 residue in M1 protein also affects its stability in H5N6 virus, we generated two M1 expression constructs derived from a chicken strain (A/goose/Shandong/F0500-14/2016, carrying A37 in M1 protein, abbreviated as F0500); one carrying A37 (H5N6-M1-37A) and the other carrying the T37 substitution (H5N6-M1-37T). H5N6-M1-37A exhibited a higher M1 protein expression level compared with H5N6-M1-37T (Figs 6A and S6A). The half-life of the H5N6-M1-37A protein was also clearly longer than that of the H5N6-M1-37T protein in both transfection (Fig 6B and 6C) and viral infection (Fig 6D and 6E). Treatment with MG132 inhibited the degradation of H5N6-M1-37T protein but had little effect on H5N6-M1-37A protein (Figs 6F and S6B). Additionally, PKG depletion in A549^Flag-PKG^ cells had no apparent effect on the protein half-life of H5N6-M1-37A (S6C and S6D Fig), but it raised the half-life of H5N6-M1-37T from 4 h to 6 h (Fig 6G and 6H), indicating that M1 protein harboring A37 in H5N6 virus shows increased protein stability as it resists degradation triggered by PKG.

To further determine the effect of the A37 mutation of M1 protein in H5N6 viral replication, we rescued the recombinant H5N6 virus derived from F0500 virus, which carried A37 (rH5N6:M1-37A) on the M1 protein, and another virus sharing the same backbone but harboring the T37 substitution (rH5N6:M1-37T). Compared with rH5N6:M1-37T virus, rH5N6:M1-37A grew to a higher titer (up to 10-fold higher, P<0.05) from 48 to 72 hpi in A549 cells (Fig 6I). Mice infected with the rH5N6:M1-37A virus showed significantly more weight loss and more severe pathological lesions than those infected with rH5N6:M1-37T virus (S6E and S6F Fig). Consistent with the pathogenicity results, the virus carrying A37 produced significantly higher titers in the lungs and turbinates compared with the rH5N6:M1-37T virus (Fig 6J and 6K). Together, these results demonstrate that the loss of the phosphorylation at T37 in H5N6 M1 protein can also increase M1 protein stability and facilitate virus replication of the emerging reassortants in human cells and in mice.

### M1 protein harboring T37 is susceptible to ubiquitination at K187

**Fig 7:**
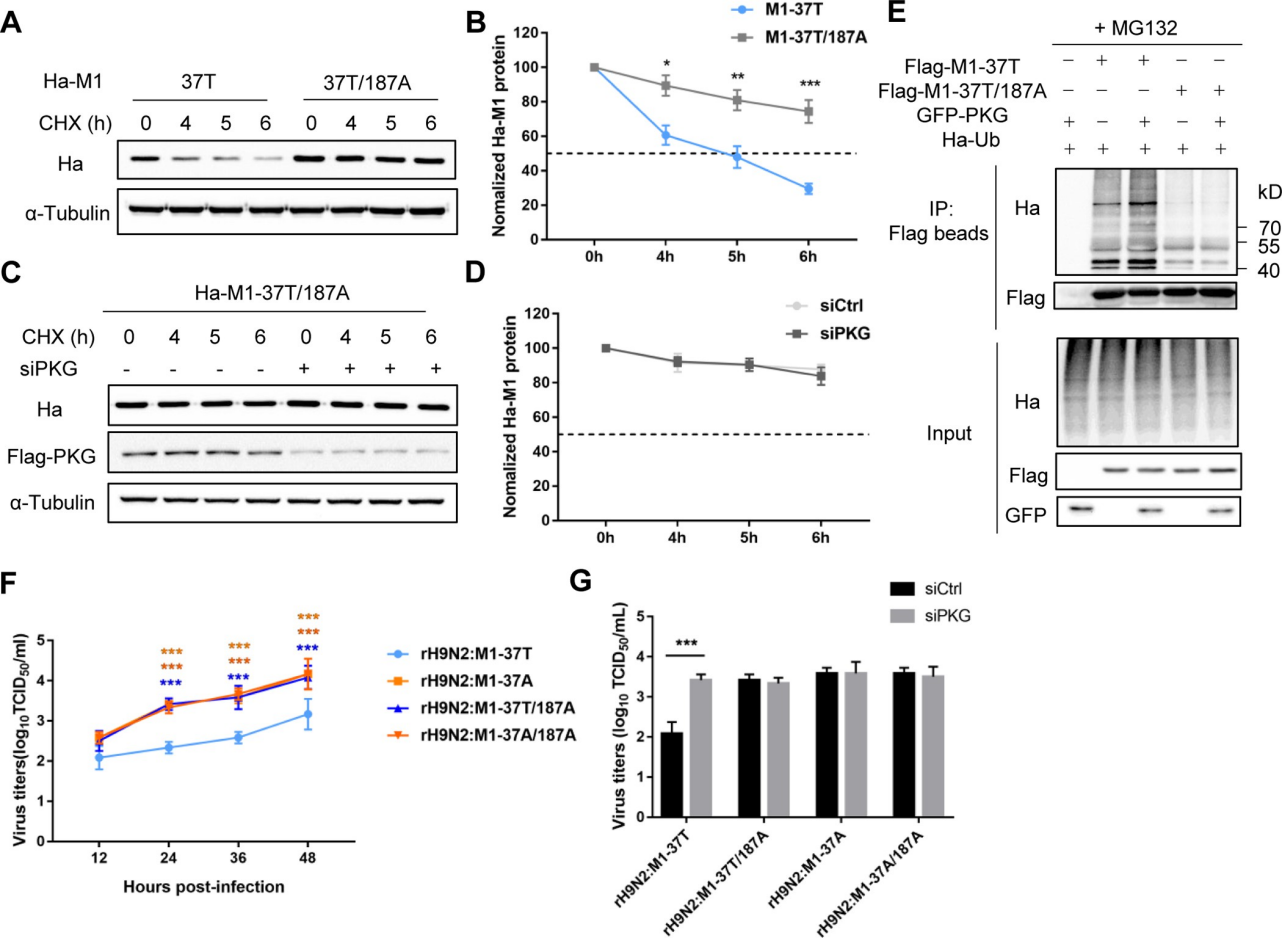
M1 protein harboring T37 is susceptible to ubiquitination at K187. (A–D) Western blotting (WB) to analyze the half-life of M1-37T and M1-37T/187A protein in A549 cells (A), and the half-life of M1-37T/187A protein in control or PKG-silenced A549^Flag-PKG^ cells (C). Cells were transfected with the indicated Ha-tagged M1 plasmids for 24 h and then treated with CHX for the indicated time. WB was performed to analyze the expression levels of Ha-M1 and Flag-PKG. α-Tubulin was used as a loading control. Densitometry analyses of the data in (A, C) was displayed in respective graphs (B, D), and the data represent the mean ± SD pooled from three independent experiments. Statistical significance was based on two-way ANOVA. The WB data are representative of three independent experiments showing similar results. (E) The effect of PKG on ubiquitination of M1 proteins. HEK293T cells were transfected with Ha-Ub plasmid, GFP-PKG, and indicated Flag-M1 or empty plasmids for 24 h and then treated with MG132 (20 μM) for 6 h. Ubiquitinated proteins were then analyzed by WB. WB results are representative of three independent experiments. (F) Virus output from infected A549 cells. A549 cells were infected with rH9N2:M1-37T, rH9N2:M1-37T/187A, rH9N2:M1-37A, or rH9N2:M1-37A/187A at a multiplicity of infection of 0.2. Virus titers were determined from supernatants collected at the indicated time points. Data are presented as the mean ± SD of three independent experiments. Statistical significance was based on two-way ANOVA (***P<0.001). (G) A549^Flag-PKG^ cells were transfected with control or PKG siRNA for 24 h and then infected by rH9N2:M1-37T, rH9N2:M1-37T/187A, rH9N2:M1-37A, or rH9N2:M1-37A/187A for 24 h. Virus titers in the collected supernatants were analyzed by determining the TCID_50_. Error bars represent the SD from the mean for three independent experiments. Statistical significance was determined by *t*-tests (***P<0.001).

To further illustrate the relationship between M1 protein stability and viral replication, we analyzed all 13 lysine (K) residues of M1 protein to identify the primary ubiquitination sites. We mutated each of these K residues to A in both M1-37T and M1-37A. Among these 13 mutants, six mutants (K47A, K57A, K187A, K230A, K242A, K252A) showed increased M1-37T protein expression levels (S7A and S7B Fig), and four mutants (K57A, K187A, K242A, K252A) showed unchanged M1-37A protein expression levels (S7C and S7D Fig). We further analyzed the effect of PKG on the expression of these M1-37T mutants. Notably, PKG depletion had little impact on the M1-37T/187A protein expression level (S8A and S8B Fig). Similarly, M1-37A/187A displayed no obvious difference in protein expression levels between the control and PKG-silenced cells (S8C and S8D Fig). Moreover, the ubiquitination levels of M1-37T/187A and M1-37A/187A were lower than that of M1-37T, suggesting that K187A might stabilize the M1 protein by preventing ubiquitination and subsequent degradation (S8E and S8F Fig). In addition, K187A increased the half-life of M1-37T protein (Fig 7A and 7B). PKG depletion had no apparent effect on the protein stability of the M1-37T or M1-37A with the K187A mutation (Figs 7C and 7D, S8G and S8H). Consistent with this, PKG overexpression enhanced the ubiquitination of M1-37T but had little effect on M1-37T/187A ubiquitination (Fig 7E). These results demonstrated that K187 is a ubiquitination site involved in PKG-mediated phosphorylation and M1 degradation.

To ascertain the function of K187 in regulating viral replication, the K187A mutation was introduced into the M gene of rH9N2:M1-37T or rH9N2:M1-37A, respectively. rH9N2:M1-37T/187A and rH9N2:M1-37A/187A viruses were rescued using a reverse genetic system. The rH9N2:M1-37T/187A, rH9N2:M1-37A, and rH9N2:M1-37A/187A viruses grew to similar titers, all of which were significantly higher than that of the rH9N2:M1-37T virus from 24 to 48 hpi in A549 cells (Fig 7F). PKG depletion had no apparent effect on the replication of rH9N2:M1-37T/187A or rH9N2:M1-37A/187A viruses at 24 hpi (Fig 7G), further indicating the K187, which is responsible for M1 stability directed by PKG, could affect the replication of virus possessing T37 in M1 protein. Together, our results confirm that improved M1 protein stability in the presence of A37 is responsible for the observed increase in viral replication.

## Discussion

In this study, we present a novel evolutionary strategy involving the M1 protein by which avian influenza virus can evade host cellular defenses. Loss of the phosphorylation at position 37 can protect M1 protein from degradation directed by PKG through a phosphorylation-dependent manner and thus contribute to enhanced M1 stability, which is considered one reason for the increased replication of avian H9N2 virus and its reassortants in humans.

For the efficient infection of human populations from avian reservoirs, AIVs must overcome the species barriers through adaptive evolution [33]. Multiple adaptive mutations in viral hemagglutinin (HA) and polymerase subunits are recognized to be necessary for the efficient infection of AIVs in their new host, even in humans [34–37]. However, few studies have reported that M1 protein is related to the increased infectivity of AIVs in mammals. Here, the phosphorylation-resistant mutation T37A of M1 protein occurred under natural selection in chickens and also exhibited a replication benefit in mice and in human cells, unlike some artificial mutations in model viruses [12,23]. As a spontaneous mutation during viral prevalence, A37 confers resistance to phosphorylation. It thereby protects M1 protein against degradation by host cellular proteins and represents a novel strategy by which AIVs establish effective replication in mammals. Our finding is supported by the presence of A37 as the dominant residue at this site of M1 protein among the recent H9N2 virus and its reassortants isolated from humans and, therefore, is of considerable clinical and epidemiological significance.

Our previous findings showed that A37 on M1 protein was first detected in 2007 and the replacement of T37 with A37 on M1 protein has been occurring in chicken H9N2 isolates since 2010. The A37 residue in the M1 protein improved viral infectivity in chickens, with an early surge in progeny virus production, more severe pathology, and extrapulmonary virus spread [22]. It was also found that the T37A mutation can increase M1 protein stability in avian DF1 cells (S9 Fig). We speculate that a similar mechanism, akin to PKG-mediated phosphorylation, operates in avian cells to control M1 protein stability and viral replication, which would support by the findings reported in our previous work. Similarly, a previous study revealed that H9N2 AIVs with an HA-A316S substitution and/or a shorter neuraminidase (NA) stalk are predominantly prevalent in avian strains, and these changes can increase the virulence in both chickens and mice [38]. Therefore, emerging or established mutations in AIVs can increase viral infectivity not only in birds but also in mammalian hosts, which may promote opportunities for interspecies transmission when humans are in contact with infected poultry or contaminated environments.

A variety of phosphorylated proteins are targeted for degradation in a proteasome-dependent manner [39]. For proteasome-mediated degradation pathways, specificity is determined by clearly defined motifs localized within the protein substrate, such as phosphorylation or dephosphorylation signals [9]. A typical example is the phosphorylation of IκB at S32 and S36 by IκB kinase, which is the signal for ubiquitination and degradation by the proteasome [25]. However, to date, no studies have reported that viral proteins of influenza virus can be degraded through a phosphorylation-dependent manner. Here, we report that the T37 residue of the M1 protein of H9N2 influenza virus can be phosphorylated by PKG, which induces the proteasomal degradation of the M1 protein. To further illustrate the relationship between M1 protein stability and viral replication, we analyzed all K residues of M1 protein to identify the primary ubiquitination sites. Our result shows that K187, whose ubiquitination is primed by PKG-mediated T37 phosphorylation, can affect the viral replication. In mammalian cells, PKGI (referred to as PKG in this paper, which has two isoforms, PKG-1α and PKG-1β) and PKG II, expressed from separate genes, mediate cGMP-dependent kinase activity [40]. PKG is a serine/threonine specific kinase and the major effector of cGMP, regulating various physiological processes in many different cell types [41]. A previous study showed that dengue virus (DENV-2) NS5 can be phosphorylated by PKG at a site conserved among all mosquito-borne flaviviruses [42]. Additionally, another cellular kinase, casein kinase 2 (CK2), was also predicted to be the kinase for T37 in M1 protein of WSN virus [23], but Jens *et al*. verified that this residue could not be phosphorylated by CK2 [43]. Interestingly, challenging the generally accepted belief that substrates with K48-linked polyubiquitin chains function as signals for proteasomal degradation, we found that ubiquitination of M1 occurred through the K63 but not the K48 linkage. A recent study reported that K63 ubiquitination could trigger proteasomal degradation by seeding branched ubiquitin chains [44]. Li *et al*. also found that TANK-binding kinase 1 (TBK1) functions as an E3 ubiquitin ligase to degrade the foot-and-mouth disease virus VP3 protein by K63 ubiquitination [45]. Our results also showed that the T37A mutation of M1 protein had no detectable effect on the viral thermostability (S10 Fig). We believe this evidence represents a novel host-AIV interaction that leads to viral M1 protein being phosphorylated and degraded in a PKG-dependent manner. We have not excluded the possibility that other unknown kinases, in addition to PKG, also act on T37 to influence M1 phosphorylation, ubiquitination and degradation. Because PKG is not an E3 ubiquitin ligase, screening for the E3 ubiquitin ligase responsible for the degradation of M1 protein and identifying the cross-link between PKG-mediated phosphorylation and ubiquitination would provide a more comprehensive understanding of our findings.

M1 protein plays multiple roles in the influenza virus life cycle. It is accepted that abundant M1 proteins are necessary for efficient replication of the virus. Liu *et al*. found that replication of influenza virus was inhibited by accelerating degradation of the M1 protein through the ubiquitin and proteasome-dependent pathway induced by CypA; however, they used the WSN virus as a model and the mechanism involved remained unclear [46]. For other viruses, although the hepatitis C virus NS2 protein can be phosphorylated by the protein kinase CK2 and targeted for degradation to the proteasome, the effect of the kinase on viral replication has not been proven [30]. Here, we demonstrate that replication of H9N2 virus carrying phosphorylation site T37 in M1 protein was restricted by kinase PKG. Thus, when T37 in M1 protein is substituted with A37, the loss of phosphorylation avoids M1 degradation and confers enhanced replication of H9N2 and H5N6 viruses. In support of our study, a previous report showed that an M1 mutant virus produced lower levels of M1 protein and propagated to significantly lower titers than wildtype virus did. Interestingly, the M1 mutant virus particles and wildtype virus particles contained the same amounts of M1 [47]. The authors proposed that a threshold amount of M1 protein is needed for virus particle assembly and that this process is delayed when the amount of M1 protein is limited. We also found that the rH9N2:M1-37T and rH9N2:M1-37A viruses have similar particle to plaque formation units (PFU) ratios (S11 Fig). Therefore, we speculate that the reduced M1-37T protein leads to a delayed assembly of viral components into infectious particles and that budding is delayed in rH9N2:M1-37T virus-infected cells until sufficient levels of M1 protein accumulate at the plasma membrane, consequently resulting in lower replication than did rH9N2:M1-37A virus. On the other hand, we found that the phosphothreonine signal of M1-37T was first detected at 4 hpi and continued to increase up to the late stage of viral infection (S12 Fig). However, position 37 is not located at, or close to, known M1 functional domains. Because PKG locates in the cytoplasm, from the viral mRNA expression level (S13 Fig), M1 transport efficiency (S14 Fig), and viral replication kinetics (Fig 2C) results, we speculate that PKG-directed M1-37T phosphorylation occurs when the newly produced M1 protein is expressed and exposed to the cytoplasm. The export of the influenza virus viral ribonucleoproteins (vRNP) to the nucleus is precisely regulated. Because M1 can inhibit the transcriptional activity of vRNP, it is expressed only in the late stage of infection, after the polymerase has completed the replication process, to avoid the premature export of vRNP to the nucleus [19]. At a later step when vRNP calls for export and transition to next phase of viral replication, more rapid expression of M1 protein (e.g., as occurs in the case of M1 with the T37A mutation) allow the threshold amount of M1 protein to be reached faster, thus facilitating the transition and shortening the viral life cycle. Such a strategy is expected to minimize the host immune response and maximize the viral production. In fact, a significant amount of viral structural elements, including genomes and structural proteins, are often made in large excess of what is packaged into infectious virions in infected cells, which may further support the essential role of such a hypothetical transition facilitated by M1. In addition, the T37 phosphorylation may not affect the virion integrity owing to decreased M1 protein stability, but it changes the morphological phenotypes of H9N2 virus. Approximately 59.80% of rH9N2:M1-37T virus particles were morphologically filamentous, whereas the rH9N2:M1-37A virions were predominantly (96.05%) spherical or ovoid (S15 Fig), which is similar to the results of our previous study [22]. Further studies will be performed to understand the role of T37 phosphorylation in the viral life cycle.

In conclusion, we reveal a novel evolutionary strategy involving the M1 protein by which AIV evades host cell defenses. This mechanism is critical for H9N2 AIV and its reassortants in combating host defenses and in adapting to the human host. It is vital to carry out continuous surveillance of such genetic variations to help prevent the emergence of potentially pandemic influenza viruses.

## Materials and methods

### Ethics statement

All animal studies were performed in compliance with the Guide for the Care and Use of Laboratory Animals of China Agricultural University (CAU) (ID: SKLAB-B-2018-021) and with approval of the Beijing Association for Science and Technology of China (approval ID SYXK, Beijing, 2007–0023).

### Viruses, plasmids and cells

The use of wild-type H9N2 viruses, A/chicken/Shandong/lx1023/2007 (lx1023) and A/chicken/Beijing/3/1999 (BJ/3/99) were described previously [22,48]. The H5N6 virus, A/goose/Shandong/F0500-14/2016 (F0500), was preserved by our laboratory, and the M gene of F0500 originated from H9N2 subtype AIVs by continued reassortment. The M1 proteins of lx1023, BJ/3/99, and F0500 possess T37, T37, and A37, respectively. In addition, the F0500 virus has an HA-Q226L mutation but does not have a PB2-E627K mutation.

The H9N2 M1 genes were amplified from lx1023 and BJ/3/99, respectively, and the H5N6 M1 gene was amplified from the M gene of F0500. The T37A mutation in the backbone of lx1023 or BJ/3/99 and the A37T mutation in the backbone of F0500 were generated by polymerase chain reaction (PCR). PKG was amplified from the mRNA of HEK293T cell. To avoid the influence of PKG on the CMV promoter [49], all M genes were cloned into the pSin vector with an EF1α promoter carrying an N-terminal Flag or Ha tag. PKG genes were cloned into a pRK5 vector with an N-terminal Flag or Ha tag or into a pEGFPC1 vector.

Human embryonic kidney (HEK293T) cells, human pulmonary adenocarcinoma (A549) cells, and Madin-Darby canine kidney (MDCK) cells were maintained in Dulbecco’s modified Eagle’s medium (DMEM, Gibco) supplemented with 10% fetal bovine serum (Gibco), 100 units/mL of penicillin, and 100 μg/mL of streptomycin at 37°C in 5% CO_2_ atmosphere.

### Reagents and antibodies

MG132 (133407-82-6) was purchased from APExBIO. ANTI-FLAG M2 Affinity Gel (Flag beads, A2220), 3×Flag peptide (F4799), and chloroquine (CQ, C6628) were purchased from Sigma-Aldrich. Cycloheximide (CHX, HY-12320) was purchased from MedChemExpress (MCE). The following antibodies were used for co-Immunoprecipitation (co-IP) and western blotting: anti-Flag (F1804, Sigma), anti-α-Tubulin (PM054, MBL), anti-HA (sc-805, Santa Cruz), anti-GFP (sc-9996, Santa Cruz), anti-influenza A virus NP (A01506, GenScript), anti-phosphothreonine (9381, Cell Signaling Technology, CST). Mouse monoclonal anti-influenza A virus M1 antibody was kindly provided by Dr. Wenjun Liu (Institute of Microbiology, Chinese Academy of Sciences, Beijing, China).

### Generation of recombinant and mutant H9N2/H5N6 viruses by reverse genetics

All eight gene segments of lx1023, BJ/3/99, or F0500 were amplified by reverse transcription (RT)-PCR and individually cloned into a dual-promoter plasmid pHW2000. The rescued viruses for lx1023, BJ/3/99, and F0500 were named rH9N2:M1-37T, rBJ/3/99:M1-37T, and rH5N6:M1-37A, respectively. The T37A mutation was introduced into the M gene of rH9N2:M1-37T or rBJ/3/99:M1-37T, and the A37T mutation was introduced into the M gene of rH5N6:M1-37A by using pairs of site-directed PCR primers. To identify the roles of K187 in M1 stability and viral replication, a K187A mutation was introduced into the M gene of rH9N2:M1-37T or rH9N2:M1-37A. The resulting mutant viruses are named as rH9N2:M1-37T/187A and rH9N2:M1-37A/187A, respectively. Primer sequences are available upon request. Reverse genetic viruses were generated in HEK293T cells as previously described and were propagated in 10-day-old specific-pathogen-free chicken embryos [50]. The titers of the stock viruses were determined by 50% tissue culture infectious dose (TCID_50_) in MDCK cells. Viral RNA was extracted and analyzed by RT-PCR, and each viral segment was sequenced by Beijing Tsingke Biological Technology to confirm the identity of the virus before use.

### Mouse challenge study

Fourteen mice (6-week-old female BALB/c mice; Boehringer Ingelheim, Beijing, China) per group were anesthetized with 20 mg/g tiletamine-zolazepam (Zoletil; Virbac SA, Carros, France) and inoculated intranasally with 50 μL of 10^6^ TCID_50_ of each virus diluted in phosphate-buffered saline (PBS). Five mice from each group were monitored daily for 14 days, and mice that lost more than 30% of their original bodyweight were humanely euthanized. Three mice from each group were euthanized at 3, 5, and 7 dpi for virus titration and histology. Lungs and nasal turbinates were collected and homogenized in 1 mL of cold PBS. Viral titers were determined by TCID_50_ assays. The virus titer detection limit is 0.75 log_10_TCID_50_/mL. A portion of the lung from each euthanized mouse at 3 dpi was fixed in 10% phosphate-buffered formalin for histopathological examination.

### Histopathology and immunohistochemistry examination

Lungs and nasal turbinates collected at 3 dpi were fixed in 10% buffered formalin, embedded in paraffin, sectioned and stained with hematoxylin and eosin (H&E). The tissue sections were then immunostained for viral nucleoprotein (NP) with a monoclonal primary antibody (A01506, GenScript). Secondary antibody (Millipore, Billerica, MA, USA) used was conjugated to horseradish peroxidase (HRP), and the color reaction was based on the use of an HRP reaction kit (diaminobenzidine-tetrahydrochloride, Sigma).

### Virus titration and replication kinetics

TCID_50_ was determined in MDCK cells with 10-fold serially diluted viruses inoculated at 37°C for 48 h. The TCID_50_ value was calculated by the Reed-Muench method. Replication kinetics was determined by inoculating A549 cells with viruses at a multiplicity of infection (MOI) of 0.2 or 1.0 for the H9N2 viruses and at an MOI of 0.001 for the H5N6 viruses. After 1 h of incubation at 37°C, the cells were washed at least twice with PBS and then further incubated in serum-free DMEM containing 1.0 μg/mL tosylsulfonyl phenylalanyl chloromethyl ketone (TPCK) trypsin. Supernatants were sampled at 12, 24, 36, 48, 60, and 72 hpi, or 2, 4, 6, 8, 10, and 12 hpi. All collected supernatants were titrated on MDCK cells in 96-well plates; three independent experiments were performed.

### Immunoprecipitation and western blotting

Immunoprecipitation of endogenous proteins was performed as described previously [51]. In brief, cells were lysed in lysis buffer (50 mM Tris-Cl at pH 8.0, 150 mM NaCl, 1% Triton X-100, 1 mM DTT, 1× complete protease inhibitor cocktail, and 10% glycerol) and pre-cleared with protein G Sepharose beads (GE Healthcare, Piscataway, NJ, USA) for 2 h at 4°C. The lysates were then immunoprecipitated with indicated antibodies or isotype-matched control antibodies plus protein G Sepharose beads at 4°C for 2–4 h. The beads were then washed three times and boiled. Protein samples were analyzed by western blotting. For the immunoprecipitation of overexpressed Flag-tagged proteins, cell lysates were incubated with Flag beads (Sigma-Aldrich) for 2–4 h at 4°C. For western blotting, cells were either directly lysed in 2× sodium dodecyl sulphate (SDS) sample buffer or extracted as described above and then mixed with SDS sample buffer. The prepared protein samples were resolved by SDS-PAGE and then transferred onto a polyvinylidene difluoride membrane. The membranes were blocked with 5% skimmed milk in PBST (PBS containing 0.5% Tween 20) for 2 h at room temperature and then incubated with specific primary antibodies overnight at 4°C, followed by an incubation with secondary antibodies for 45 min at room temperature. The reactive protein bands were visualized using an enhanced chemiluminescence reagent with a Tanon-5200 luminescent imaging workstation.

### Establishment of stable A549 cell lines overexpressing H9N2 M1 protein

HEK293T cells cultured in 10-cm dishes were transfected either with the retroviral construct pSin-M1-37T or pSin-M1-37TA, or with the empty pSin vector, together with two lentivirus packaging plasmids, pMD2.G and psPAX2, by using jetPRIME (Polyplus-transfection, NY, USA), and the pSin/psPAX2/pMD2.G ratio was 2:2:1. At 48 h post-transfection, viral supernatants were collected, filtered through a 0.45-μm syringe filter, and then used to transduce A549 cells cultured in 6-well plates in the presence of 8 μg/mL Polybrene. The confluent transduced cells were split and cultured in medium supplemented with 2.5 μg/mL puromycin for selection for a week. The surviving cells were propagated, and examined for M1 expression by sequencing and western blotting.

### Generation of a A549 cell line that endogenously expresses Flag-PKG (A549^Flag-PKG^)

To facilitate the affinity purification of PKG, we constructed an A549 cell line that endogenously expresses Flag-PKG by adding *Flag* to the *PKG* gene using the CRISPR-Cas9 technique. To add *Flag* to the endogenous *PKG*, A549 cells were seeded into a 6-well dish to achieve 70% confluency and were transfected with CRISPR/Cas9 plasmids containing a target sequence complementary to the intron that was prior to the ATG of PKG plus a donor plasmid containing homologous arms and Puro-P2A-3×Flag sequences. After 48 h, medium containing 2.5 mg/mL puromycin was added to select for tagged cells, and a further 48 h later, the cells were diluted and seeded into a 96-well dish at 0.5 cell/well in complete DMEM media. Wells that contained a single colony each were expanded until enough cells were available for total protein extraction to determine the presence of Flag-PKG by western blotting. Primers used for encoding sgRNA for Cas9 targeting the intron of PKG to generate A549^Flag-PKG^ cells are: PKG-Intron-F: CACCGGGCGCTAAGTACTCGAGCG, PKG-Intron-R: AAACCGCTCGAGTACTTAGCGCCCC.

### Protein degradation assay

A549 or A549^Flag-PKG^ cell lines were transfected with M1 plasmids, or infected with H9N2 or H5N6 viruses. After 24 h post-transfection or infection, CHX (50 μg/mL) was added to the medium, and the cells were harvested at different time points. Western blotting was performed on cell lysates to detect M1, NP, α-Tubulin, and Flag-PKG. To investigate the cellular pathway mediating M1 degradation, at 24 h post-transfection, MG132 (20 μM) or CQ (150 μM) was added to the medium, and the cells were harvested 6 h after treatment. Three independent experiments were performed.

### Ubiquitination assay

HEK293T cells were transfected with Ha-tagged ubiquitin (Ha-Ub) plasmids, Flag-M1 or empty plasmids, and GFP-PKG. At 24 h post-transfection, the cells were treated with MG132 (20 μM) for 6 h and then lysed in 1% SDS lysis buffer. After being boiled for 10 min, the lysates were diluted 10 times with cold lysis buffer supplemented with 1× complete protease inhibitor cocktail, 10 mM DTT, and 10 mM NEM. M1 protein was then purified with Flag beads (Sigma-Aldrich) and subjected to western blotting analysis. Ubiquitinated M1 was detected by anti-Ha antibody.

### *In vitro* kinase assay

PKG was purified from overexpressed HEK293T cells by immunoprecipitation using Flag beads (Sigma-Aldrich). Immunoprecipitate was used in *in vitro* phosphorylation assay. GST-fused M1 was expressed in *Escherichia coli* strain BL21 and purified with Sepharose 4B-glutathione (GE Healthcare). The mixtures were incubated for 1 h at 30°C in a total volume of 50 μL of kinase buffer (#9802, CST), 5 mM of cGMP (G6129, Sigma-Aldrich), and 200 μM ATP (#9804, CST). The reactions were stopped by addition of 10 μL of 6× SDS loading buffer. Phosphorylated M1 was resolved by 10% SDS-PAGE and visualized by p-Thr antibodies (CST).

### RNA interference

siRNAs against PKG were transfected using Lipofectamine RNAiMax (Invitrogen) at a final concentration of 20 nM following the manufacturer’s instructions. siRNAs against PKG (5′-GAGGAAGACUUUGCCAAGAUUCUCA-3′) are constructed by GenePharma Corporation (Shanghai, China).

### PKG activity assay

A549 cells in 100-mm-dishes were infected with indicated viruses at an MOI of 2. Cells were harvested and resuspended with the suggested extraction buffer following the manufacturer’s instructions and then lysed using sonication. A PKG activity assay kit from Cyclex (Cat #CY-1161, MBL, Japan) was used to determine the relative PKG activities following the manufacturer’s instructions as previously described [52].

### ImageJ quantification, statistical analysis and reproducibility

ImageJ software was used to perform the densitometry analyses. All statistical analyses were performed using GraphPad Prism software version 7.00 (GraphPad Software Inc., San Diego, CA, USA). Statistically significant differences between experimental groups were analyzed by two-way ANOVA or *t*-tests. Survival analyses were performed using the Kaplan–Meier method and assessed using the log-rank test. Data are presented as the mean ± standard deviation (SD). Differences were considered statistically significant at *P*<0.05 (*P<0.05, **P<0.01, and ***P<0.001).

## Supporting information

S1 Fig M1-37T protein is degraded in a lysosome-independent manner, and its ubiquitination is not through K48 linkage. (A, C, D) Densitometry analyses of the data displayed in Fig 3A, Fig 3H, and Fig 3I, respectively. The data represent the mean ± SD pooled from three independent experiments. Statistical significance was based on *t*-tests (**P<0.01; ***P<0.001). (B) mRNA levels of M1-37T and M1-37A in CHX-treated A549 cells. A549 cells were transfected with Ha-tagged M1 (37T or 37A) expression plasmids for 24 h, and then treated with CHX (50 μg/mL) over indicated time course. mRNA expression of M1 genes at the indicated time points was detected by real-time PCR. mRNA expression levels are presented as fold changes relative to the values before CHX treatment. Data represent the mean ± standard deviations of results from three independent experiments. Statistical significance was based on two-way ANOVA. (E) Western blotting (WB) analysis of the expression levels of M1 in A549 cells transfected with Flag-tagged M1 (37T or 37A) plasmids for 24 h, followed by NH_4_Cl (10 mM) treatment for 6 h. α-Tubulin was used as a loading control. Densitometry analysis of the data presented in (E) is displayed in graph (F), and the data represent the mean ± SD pooled from three independent experiments. Statistical significance was based on *t*-tests. (G) HEK293T cells were co-transfected with Flag-tagged M1 (37T or 37A) or empty vector plasmid, and a plasmid encoding a version of ubiquitin capable of binding the substrate only through K48, for 24 h, followed by MG132 (20 μM) treatment for 6 h. Ubiquitinated proteins were then analyzed by WB. WB data are representative of three independent experiments showing similar results.
(TIF)

S2 Fig The T37A substitution drastically reduces the level of total M1 threonine phosphorylation. (A) Densitometry analysis of the relative threonine-phosphorylated M1 levels shown in Fig 4A. (B) Densitometry analysis of the relative threonine-phosphorylated M1 levels shown in Fig 4D. The relative ^p^T M1 intensity was determined as the ratio of threonine-phosphorylated M1 to total M1. The data represent the mean ± SD pooled from three independent experiments. Statistical significance was based on *t*-tests (**P<0.01; ***P<0.001).
(TIF)

S3 Fig The replacement of T37 with A37 protects M1 protein from degradation directed by PKG. (A, B) Densitometry analysis of the relative M1 abundance in Fig 5H and Fig 5J, respectively. The data represent the mean ± SD pooled from three independent experiments. Statistical significance was based on *t*-tests (**P<0.01). (C) Detection of PKG activity. A549 cells in 100-mm-dishes were infected with rH9N2:M1-37T or rH9N2:M1-37A at an MOI of 2. Cells were harvested at 10 h post-infection and resuspended with the suggested extraction buffer and lysed using sonication. Relative PKG activity was detected using a cGK assay kit in accordance with the manufacturer’s instruction. The data represent the mean ± SD pooled from three independent experiments. Statistical significance was based on *t*-tests. (D) PKG expression during viral infection. A549 cells were infected with rH9N2:M1-37T or rH9N2:M1-37A at an MOI of 2. Cells were harvested at the indicated time points, and Western blotting (WB) was performed on cell lysates. α-Tubulin was used as a loading control. The WB results are representative of three independent experiments.
(TIF)

S4 Fig Stability of the H9N2 M1 protein of BJ/3/99 virus is increased by the T37A mutation. (A) The protein abundance levels of BJ-M1-37T and BJ-M1-37A at different transfection doses. HEK293T cells were transfected with the indicated dose of Ha-tagged M1 (BJ-M1-37T or BJ-M1-37A). Western blotting (WB) was performed to analyze the expression levels of Ha-M1. α-Tubulin was used as a loading control. Densitometry analysis of the data presented in (A) is displayed in graph (B), and the data represent the mean ± SD pooled from three independent experiments. Statistical significance was based on *t*-tests (**P<0.01; ***P<0.001). (C–F) Protein degradation assay of BJ/3/99 M1 protein. A549 cells were transfected with Ha-tagged M1 (BJ-M1-37T or BJ-M1-37A) expression plasmids (C), or were infected with rBJ/3/99:M1-37T or rBJ/3/99:M1-37A for 24 h (E), followed by CHX (50 μg/mL) treatment over the indicated time course. Densitometry analysis of the data presented in (C, E) are displayed in respective graphs (D, F), and the data represent the mean ± SD pooled from three independent experiments. Statistical significance was based on two-way ANOVA (**P<0.01; ***P<0.001). (G–J) WB analysis to detect the expression levels of M1 in A549 cells transfected with Ha-tagged BJ-M1 (BJ-M1-37T or BJ-M1-37A) plasmids for 24 h, followed by MG132 (20 μM, G) or CQ (150 μM, I) treatment for 6 h. α-Tubulin was used as a loading control. Densitometry analysis of the data presented in (G, I) are displayed in respective graphs (H, J), and the data represent the mean ± SD pooled from three independent experiments. Statistical significance was based on *t*-tests (***P<0.001). (K) Ubiquitination analysis of BJ/3/99 M1 treated with MG132. Ha-Ub plasmid was co-transfected with Flag-tagged M1 (BJ-M1-37T or BJ-M1-37A) or empty vector plasmid into HEK293T cells for 24 h, after which the cells were treated with MG132 (20 μM) for 6 h. Ubiquitinated proteins were then analyzed by WB. All WB results are representative of three independent experiments.
(TIF)

S5 Fig T37A mutation protects M1 protein of BJ/3/99 virus from degradation directed by PKG and increases the viral replication. (A) Immunoblotting (IB) analysis of the threonine phosphorylation levels of M1 of BJ/3/99 virus. HEK293T cells transfected with Flag-tagged BJ/3/99 M1 (37T or 37A) expression plasmids or empty vector (Flag-EV) plasmid for 24 h, followed by MG132 (20 μM) treatment for 6 h, were immunoprecipitated with Flag beads. Threonine phosphorylation levels of M1 were detected by an anti-phosphothreonine antibody. α-Tubulin was used as a loading control. (B-E) Western blotting (WB) analysis of the half-life of BJ/3/99 M1 protein in control or PKG-silenced A549^Flag-PKG^ cells. Control or PKG-silenced A549^Flag-PKG^ cells were transfected with Ha-tagged BJ-M1-37T (B) or BJ-M1-37A (D) plasmid for 24 h, then treated with CHX for the indicated time. WB was performed to analyze the expression levels of Ha-M1 and Flag-PKG. Data were quantified as the ratio of Ha-M1 to α-Tubulin and were displayed in respective graphs (C, E), and the data represent the mean ± SD pooled from three independent experiments. Statistical significance was based on two-way ANOVA (**P<0.01; ***P<0.001). All WB data are representative of three independent experiments showing similar results. (F) Virus output of rBJ/3/99:M1-37T and rBJ/3/99:M1-37A viruses from infected A549 cells. A549 cells were infected at an MOI of 0.4. Virus titers were determined from supernatants collected at the indicated time points. Data are presented as the mean ± SD of three independent experiments. Statistical significance was based on two-way ANOVA (*P<0.05; **P<0.01; ***P<0.001). (G) Body weight changes in mice over a 14-day period. Six-week-old female BALB/c mice (n = 5 per group in one independent experiment) were individually inoculated with 10^6^ TCID_50_ of rBJ/3/99:M1-37T or rBJ/3/99:M1-37A or were mock infected with PBS. Each data point represents the mean ± SD and is representative of three independent experiments. Statistical significance was based on two-way ANOVA (*P<0.05; **P<0.01). (H, I) Virus titers recovered from mouse lungs and turbinates. Lungs (H) and turbinates (I) were harvested from three mice per group at 3, 5, and 7 dpi for virus titration. Each data point represents the mean ± SD and is representative of three independent experiments. Dashed lines indicate the lower limit of detection. Statistical significance was based on two-way ANOVA (*P<0.05; **P<0.01).
(TIF)

S6 Fig A37 increases the M1 protein stability and viral pathogenicity of H5N6 virus. (A, B) Densitometry analysis of the data presented in Fig 6A and Fig 6F, respectively. The data represent the mean ± SD pooled from three independent experiments. Statistical significance was based on *t*-tests (**P<0.01; ***P<0.001). (C, D) Western blotting (WB) analysis of the half-life of M1 protein in control or PKG-silenced A549^Flag-PKG^ cells. Control or PKG-silenced A549^Flag-PKG^ cells were transfected with Ha-tagged H5N6-M1-37A plasmid for 24 h and then treated with CHX (50 μg/mL) for the indicated time. WB was performed to analyze the expression levels of Ha-M1 and Flag-PKG. Data are quantified as the ratio of Ha-M1 to α-Tubulin and are displayed in graph (D). The data represent the mean ± SD pooled from three independent experiments. Statistical significance was based on two-way ANOVA. The WB data are representative of three independent experiments showing similar results. (E) Bodyweight changes in mice over a 14-day period. Six-week-old female BALB/c mice (n = 5 per group in one independent experiment) were individually inoculated with 10^6^ TCID_50_ of rH5N6:M1-37T or rH5N6:M1-37A viruses or were mock infected with PBS. Each data point represents the mean ± SD and is representative of three independent experiments. Statistical significance was based on two-way ANOVA (*P<0.05; **P<0.01). (F) Representative HE and IHC examination of lung sections from three BALB/c mice infected with 10^6^ TCID_50_ of rH5N6:M1-37T and rH5N6:M1-37A viruses at 3 dpi. Scale bar, 200 μm.
(TIF)

S7 Fig Identification of lysine (K) residues that mediate M1 stability. (A) A549 cells were transfected to express the Ha-tagged M1-37T, M1-37A, or indicated K mutants of M1-37T, respectively. Western blotting (WB) was used to detect the M1 expression levels using an anti-Ha antibody. (B) Densitometry analysis of the data presented in (A). (C) Mutations that conferred significantly increased M1-37T expression in S7A Fig were introduced into M1-37A. HEK293T cells were transfected to express the Ha-tagged M1-37T, M1-37A, or indicated K mutants of M1-37A, respectively. WB was used to detect the expression levels of M1 in A549 cells using an anti-Ha antibody. (D) Densitometry analysis of the data presented in (C). α-Tubulin was used as a loading control. All data represent the mean ± SD pooled from three independent experiments.
(TIF)

S8 Fig K187 is responsible for the M1 stability directed by PKG. (A) Western blotting (WB) analysis of Ha-M1 and Flag-PKG in control or PKG-silenced A549^Flag-PKG^ cells transfected with plasmids for Ha-tagged M1-37T, M1-37A, or the indicated mutants of M1-37T. (B) Densitometry analysis of the data presented in (A). (C) WB analysis of Ha-M1 and Flag-PKG in control or PKG-silenced A549^Flag-PKG^ cells transfected with plasmids for Ha-tagged M1-37T, M1-37A, or M1-37A/187A. α-Tubulin was used as a loading control. (D) Densitometry analysis of the data presented in (C). All data represent the mean ± SD pooled from three independent experiments. Statistical significance was based on *t*-tests (*P<0.05; **P<0.01; ***P<0.001). (E, F) Ubiquitination analysis of M1-37T mutants (E) or M1-37A mutants (F). Ha-tagged ubiquitin (Ha-Ub) plasmid was co-transfected with the indicated Flag-tagged M1 plasmid or empty vector (EV) plasmid into HEK293T cells for 24 h, followed by MG132 (20 μM) treatment for 6 h. Ubiquitinated proteins were then analyzed by WB. (G) WB analysis of the half-life of M1-37A/187A protein in control or PKG-silenced A549^Flag-PKG^ cells. Control or PKG-silenced A549^Flag-PKG^ cells were transfected with Ha-tagged M1-37A/187A plasmids for 24 h and then treated with CHX for the indicated time. WB was performed to analyze the expression levels of Ha-M1 and Flag-PKG. Densitometry analysis of the data presented in Fig G is displayed in graph (H), and the data represent the mean ± SD pooled from three independent experiments. Statistical significance was based on two-way ANOVA. All WB data are representative of three independent experiments showing similar results.
(TIF)

S9 Fig Stability of H9N2 M1 protein in DF1 cells. (A) The M1 protein abundance levels at different transfection doses in DF1 cells. DF1 cells were transfected with the indicated amounts of plasmids containing Ha-tagged M1 (37T or 37A). The Ha-M1 protein was determined by immunoblotting analysis with α-Tubulin used as a loading control. Densitometry analysis of the data presented in (A) are displayed in graph (B), and the data represent the mean ± SD pooled from three independent experiments. Statistical significance was based on *t*-tests (*P<0.05). (C-F) Protein degradation assay of M1 protein in DF1 cells. DF1 cells were transfected with Ha-tagged M1 (37T or 37A) expression plasmids for 24 h (C), or were infected with rH9N2:M1-37T or rH9N2:M1-37A for 24 h (E), followed by CHX (50 μg/mL) treatment over the indicated time course. NP protein was used as a viral protein control. The half-life of M1 or NP proteins (normalized to α-Tubulin) as determined by western blotting is displayed in the respective graphs. Densitometry analysis of the data presented in (C, E) are displayed in respective graphs (D, F), and the data represent the mean ± SD pooled from three independent experiments. Statistical significance was based on two-way ANOVA (*P<0.05; **P<0.01; ***P<0.001).
(TIF)

S10 Fig Effect of the M1-T37A substitution on viral thermostability. Viruses were adjusted to 7 log2 HA units. The thermal stability was assessed by examining the ability of each virus to hemagglutinate chicken erythrocytes after incubation at the indicated temperatures for 45 min (A) or at 56°C for the time course up to 240 minutes (B). The data are representative of three independent experiments.
(TIF)

S11 Fig The particle to PFU ratio of rH9N2:M1-37T and rH9N2:M1-37A. The viral particle to PFU ratio was determined as previously described [53]. Briefly, RNA was extracted and purified from each culture supernatant, and the viral RNA copy number in the supernatant was determined by qRT-PCR and regarded as the number of virus particles. A virus titer in PFU of each culture supernatant was also determined by plaque assay, and particle-to-PFU ratio was calculated. The particle to PFU ratio of the rH9N2:M1-37T and rH9N2:M1-37A are 26.3±0.64 and 29.3±1.60, respectively.
(TIF)

S12 Fig Phosphothreonine levels of the M1 proteins in A549 cells during viral infection. A549 cells were infected with the rH9N2:M1-37T or rH9N2:M1-37A H9N2 viruses at an MOI of 10 for 1–9 h. Cells were then harvested and lysed at the indicated times post infection, and the threonine phosphorylation levels of M1 were detected by anti-phosphothreonine antibody. Western blotting analyzed the expression levels of M1 and NP, and β-Actin was used as loading control.
(TIF)

S13 Fig Relative expression levels of viral M1, M2, and PA mRNAs of the rH9N2:M1-37T and rH9N2:M1-37A H9N2 viruses in A549 cells. A549 cells were infected with the indicated H9N2 viruses at an MOI of 1 for 0, 2, 4, 6 and 12 h or at an MOI of 0.2 for 24 h. The mRNA expression levels are presented as fold changes relative to the values for the rH9N2:M1-37T virus. Data are presented as mean ± standard deviations of results from three independent experiments. Statistical significance was based on two-way ANOVA (***P<0.001).
(TIF)

S14 Fig Subcellular localization of M1 protein in A549 cells. A549 cells were infected at an MOI of 2 with rH9N2:M1-37T or rH9N2:M1-37A. Cells were harvested at 4, 6, 8 hpi. The nuclear and cytoplasmic fractions were prepared using a Nuclear and Cytoplasmic Protein Extraction Kit (Beyotime Biotechnology, Shanghai, China) according to the manufacturer’s instructions. Lysates were analyzed by immunoblotting with antibodies to M1, NP, α-Tubulin and Histone H3. These results represent three independent experiments.
(TIF)

S15 Fig Transmission electron micrographs of negatively stained H9N2 virus particles harboring the T37 (A) or A37 (B) in M1 protein. For imaging of virions, samples were prepared as previously described [22]. Cells were infected at an MOI of 3.0 for 15 h. rH9N2:M1-37T progeny were mainly filamentous, and rH9N2:M1-37A particles were primarily spherical/ovoid.
(TIF)
