# Supplementary figures and images for: Enhanced stability of M1 protein mediated by a phospho-resistant mutation promotes the replication of prevailing avian influenza virus in mammals
Source: PLoS Pathog. 2022 Jul 6;18(7):e1010645. doi: 10.1371/journal.ppat.1010645 (PMC9258882; doi:10.1371/journal.ppat.1010645)

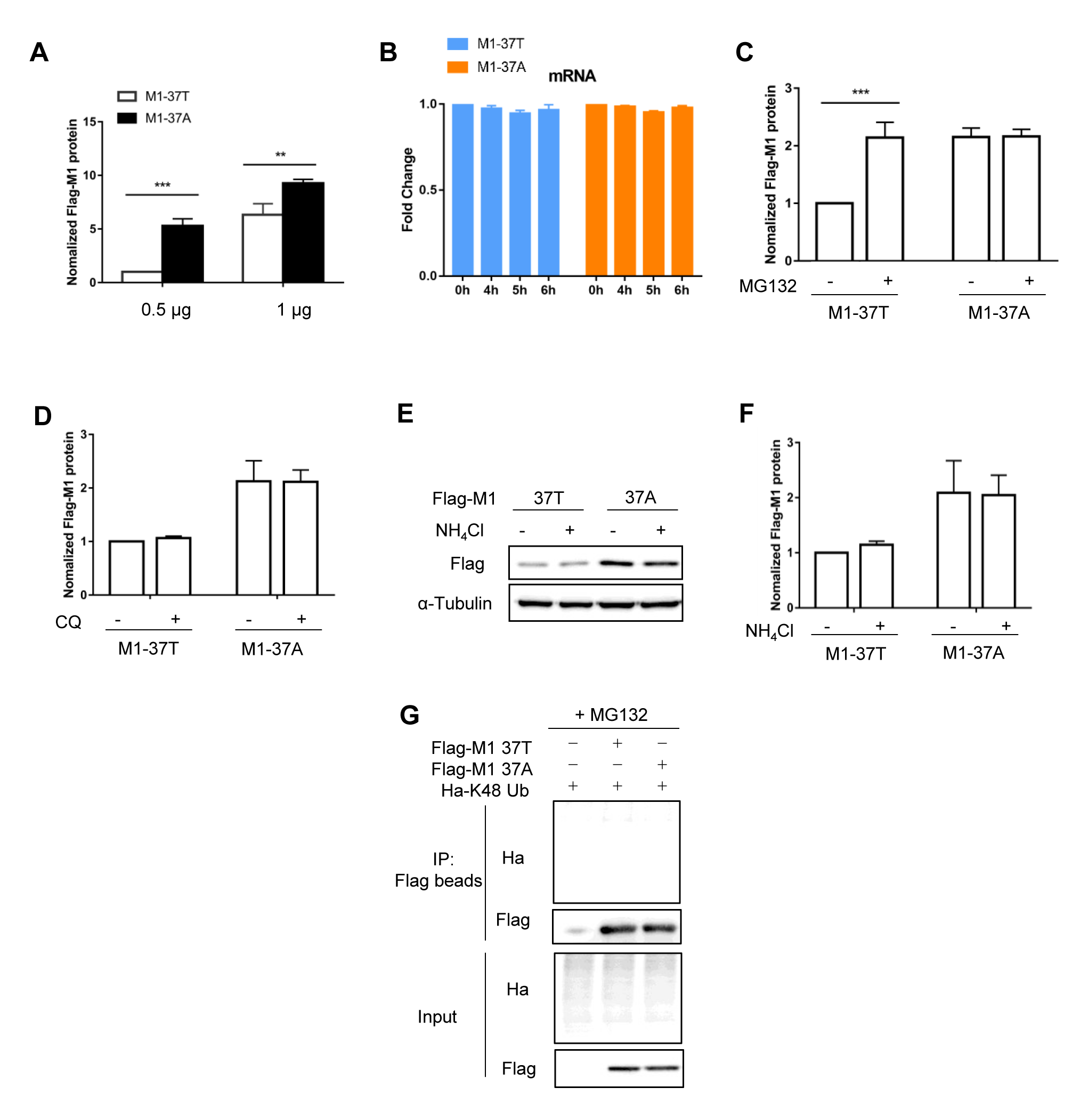

Supplement: S1 Fig — (A, C, D) Densitometry analyses of the data displayed in Fig 3A, Fig 3H, and Fig 3I, respectively. The data represent the mean ± SD pooled from three independent experiments. Statistical significance was based on t-tests (**P<0.01; ***P<0.001). (B) mRNA levels of M1-37T and M1-37A in CHX-treated A549 cells. A549 cells were transfected with Ha-tagged M1 (37T or 37A) expression plasmids for 24 h, and then treated with CHX (50 μg/mL) over indicated time course. mRNA expression of M1 genes at the indicated time points was detected by real-time PCR. mRNA expression levels are presented as fold changes relative to the values before CHX treatment. Data represent the mean ± standard deviations of results from three independent experiments. Statistical significance was based on two-way ANOVA. (E) Western blotting (WB) analysis of the expression levels of M1 in A549 cells transfected with Flag-tagged M1 (37T or 37A) plasmids for 24 h, followed by NH4Cl (10 mM) treatment for 6 h. α-Tubulin was used as a loading control. Densitometry analysis of the data presented in (E) is displayed in graph (F), and the data represent the mean ± SD pooled from three independent experiments. Statistical significance was based on t-tests. (G) HEK293T cells were co-transfected with Flag-tagged M1 (37T or 37A) or empty vector plasmid, and a plasmid encoding a version of ubiquitin capable of binding the substrate only through K48, for 24 h, followed by MG132 (20 μM) treatment for 6 h. Ubiquitinated proteins were then analyzed by WB. WB data are representative of three independent experiments showing similar results. (TIF) [file ppat.1010645.s001.tif]

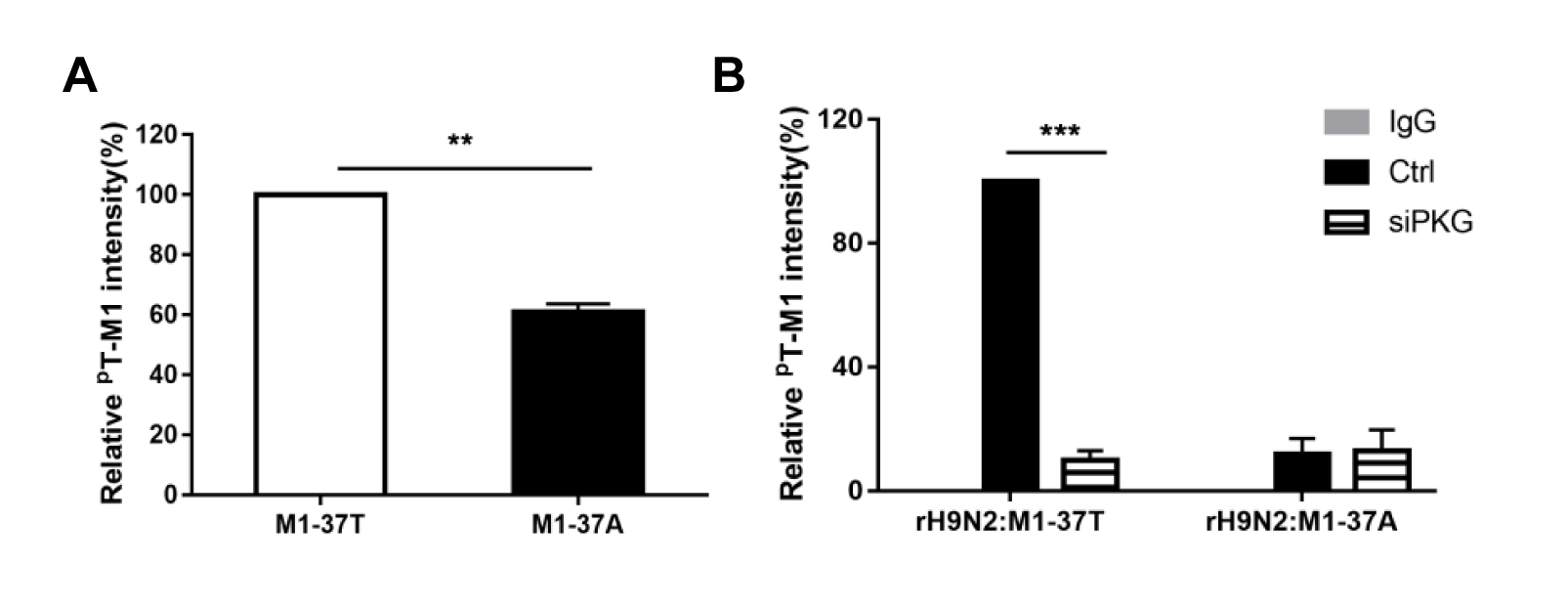

Supplement: S2 Fig — (A) Densitometry analysis of the relative threonine-phosphorylated M1 levels shown in Fig 4A. (B) Densitometry analysis of the relative threonine-phosphorylated M1 levels shown in Fig 4D. The relative pT M1 intensity was determined as the ratio of threonine-phosphorylated M1 to total M1. The data represent the mean ± SD pooled from three independent experiments. Statistical significance was based on t-tests (**P<0.01; ***P<0.001). (TIF) [file ppat.1010645.s002.tif]

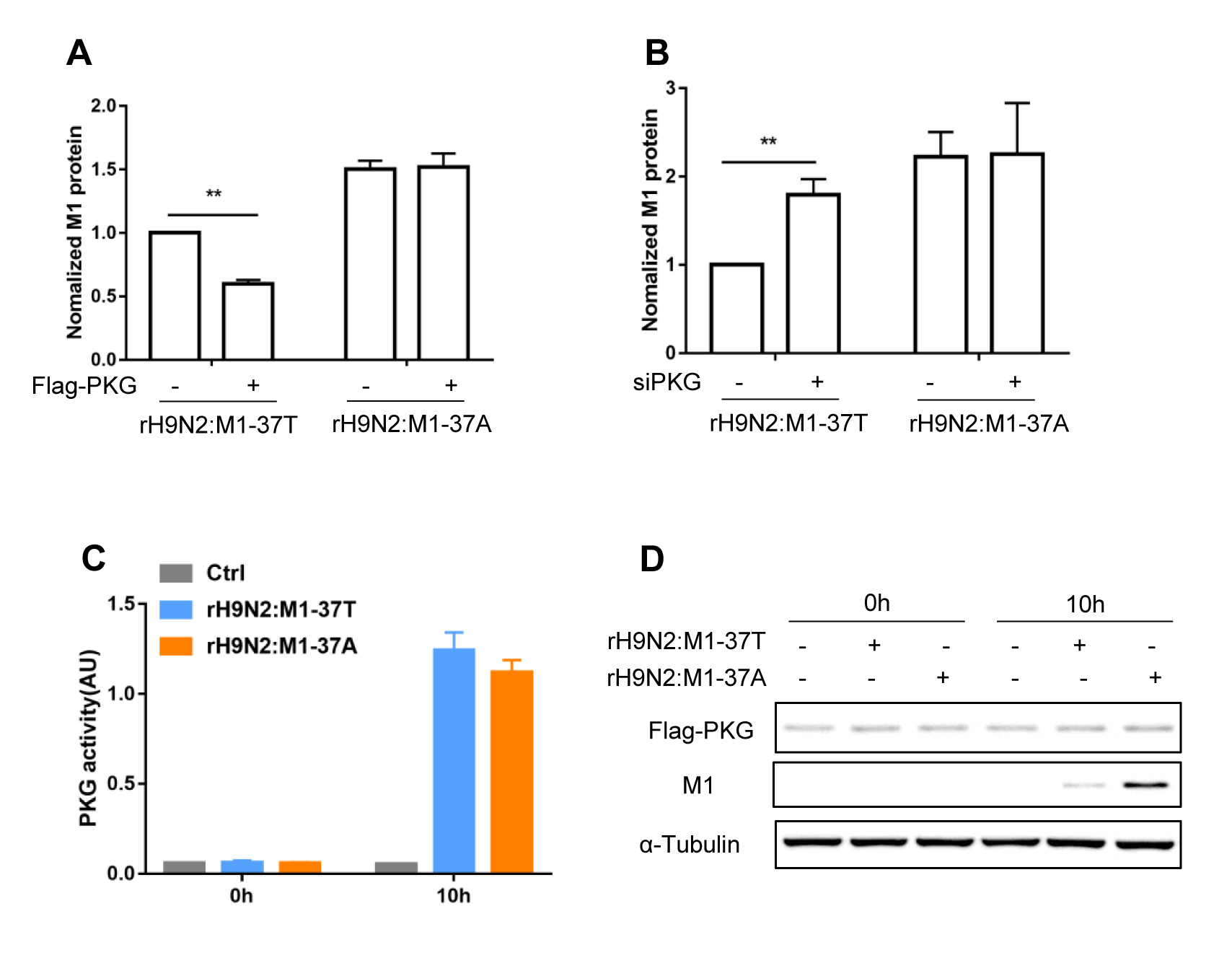

Supplement: S3 Fig — (A, B) Densitometry analysis of the relative M1 abundance in Fig 5H and Fig 5J, respectively. The data represent the mean ± SD pooled from three independent experiments. Statistical significance was based on t-tests (**P<0.01). (C) Detection of PKG activity. A549 cells in 100-mm-dishes were infected with rH9N2:M1-37T or rH9N2:M1-37A at an MOI of 2. Cells were harvested at 10 h post-infection and resuspended with the suggested extraction buffer and lysed using sonication. Relative PKG activity was detected using a cGK assay kit in accordance with the manufacturer’s instruction. The data represent the mean ± SD pooled from three independent experiments. Statistical significance was based on t-tests. (D) PKG expression during viral infection. A549 cells were infected with rH9N2:M1-37T or rH9N2:M1-37A at an MOI of 2. Cells were harvested at the indicated time points, and Western blotting (WB) was performed on cell lysates. α-Tubulin was used as a loading control. The WB results are representative of three independent experiments. (TIF) [file ppat.1010645.s003.tif]

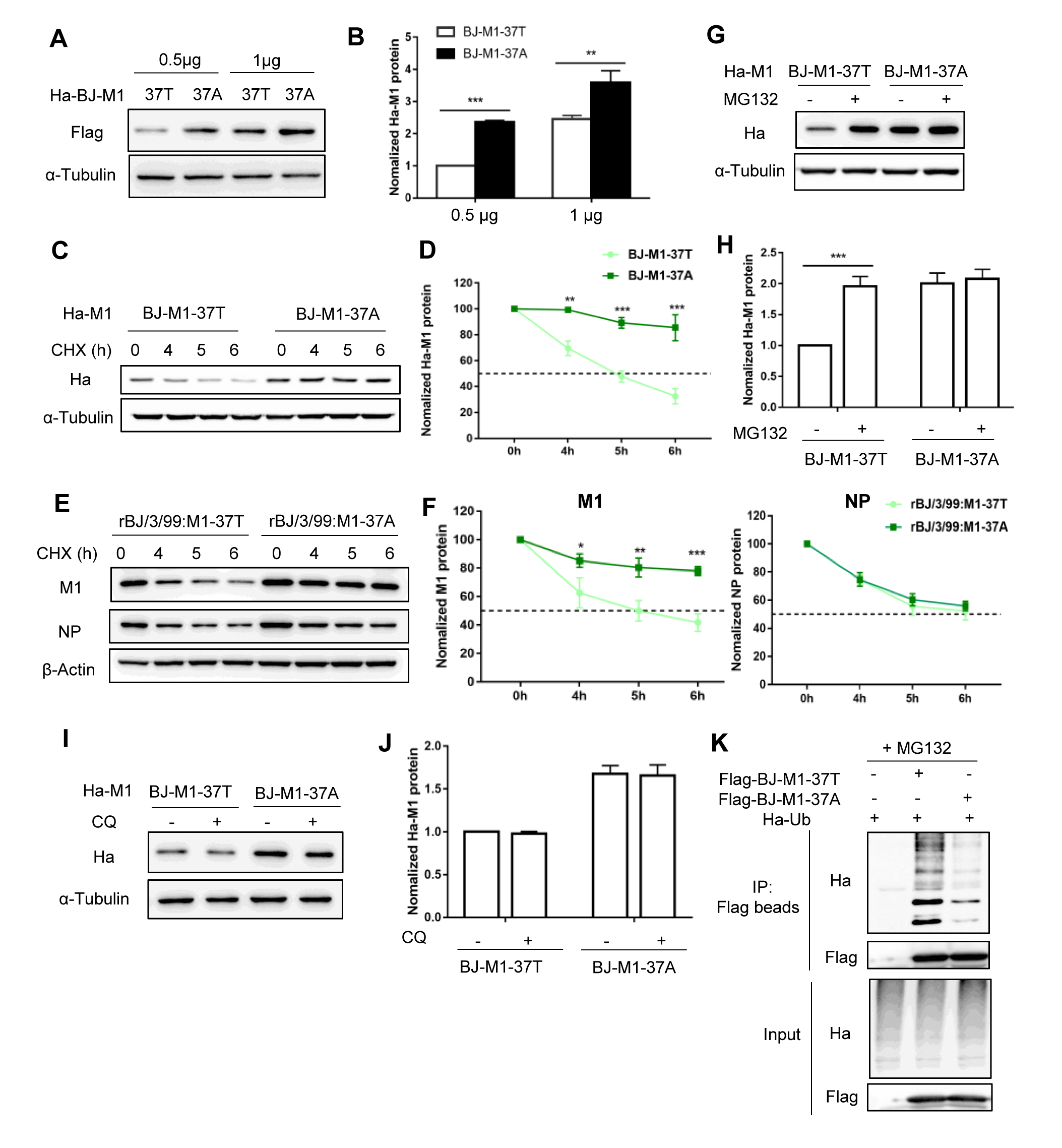

Supplement: S4 Fig — (A) The protein abundance levels of BJ-M1-37T and BJ-M1-37A at different transfection doses. HEK293T cells were transfected with the indicated dose of Ha-tagged M1 (BJ-M1-37T or BJ-M1-37A). Western blotting (WB) was performed to analyze the expression levels of Ha-M1. α-Tubulin was used as a loading control. Densitometry analysis of the data presented in (A) is displayed in graph (B), and the data represent the mean ± SD pooled from three independent experiments. Statistical significance was based on t-tests (**P<0.01; ***P<0.001). (C–F) Protein degradation assay of BJ/3/99 M1 protein. A549 cells were transfected with Ha-tagged M1 (BJ-M1-37T or BJ-M1-37A) expression plasmids (C), or were infected with rBJ/3/99:M1-37T or rBJ/3/99:M1-37A for 24 h (E), followed by CHX (50 μg/mL) treatment over the indicated time course. Densitometry analysis of the data presented in (C, E) are displayed in respective graphs (D, F), and the data represent the mean ± SD pooled from three independent experiments. Statistical significance was based on two-way ANOVA (**P<0.01; ***P<0.001). (G–J) WB analysis to detect the expression levels of M1 in A549 cells transfected with Ha-tagged BJ-M1 (BJ-M1-37T or BJ-M1-37A) plasmids for 24 h, followed by MG132 (20 μM, G) or CQ (150 μM, I) treatment for 6 h. α-Tubulin was used as a loading control. Densitometry analysis of the data presented in (G, I) are displayed in respective graphs (H, J), and the data represent the mean ± SD pooled from three independent experiments. Statistical significance was based on t-tests (***P<0.001). (K) Ubiquitination analysis of BJ/3/99 M1 treated with MG132. Ha-Ub plasmid was co-transfected with Flag-tagged M1 (BJ-M1-37T or BJ-M1-37A) or empty vector plasmid into HEK293T cells for 24 h, after which the cells were treated with MG132 (20 μM) for 6 h. Ubiquitinated proteins were then analyzed by WB. All WB results are representative of three independent experiments. (TIF) [file ppat.1010645.s004.tif]

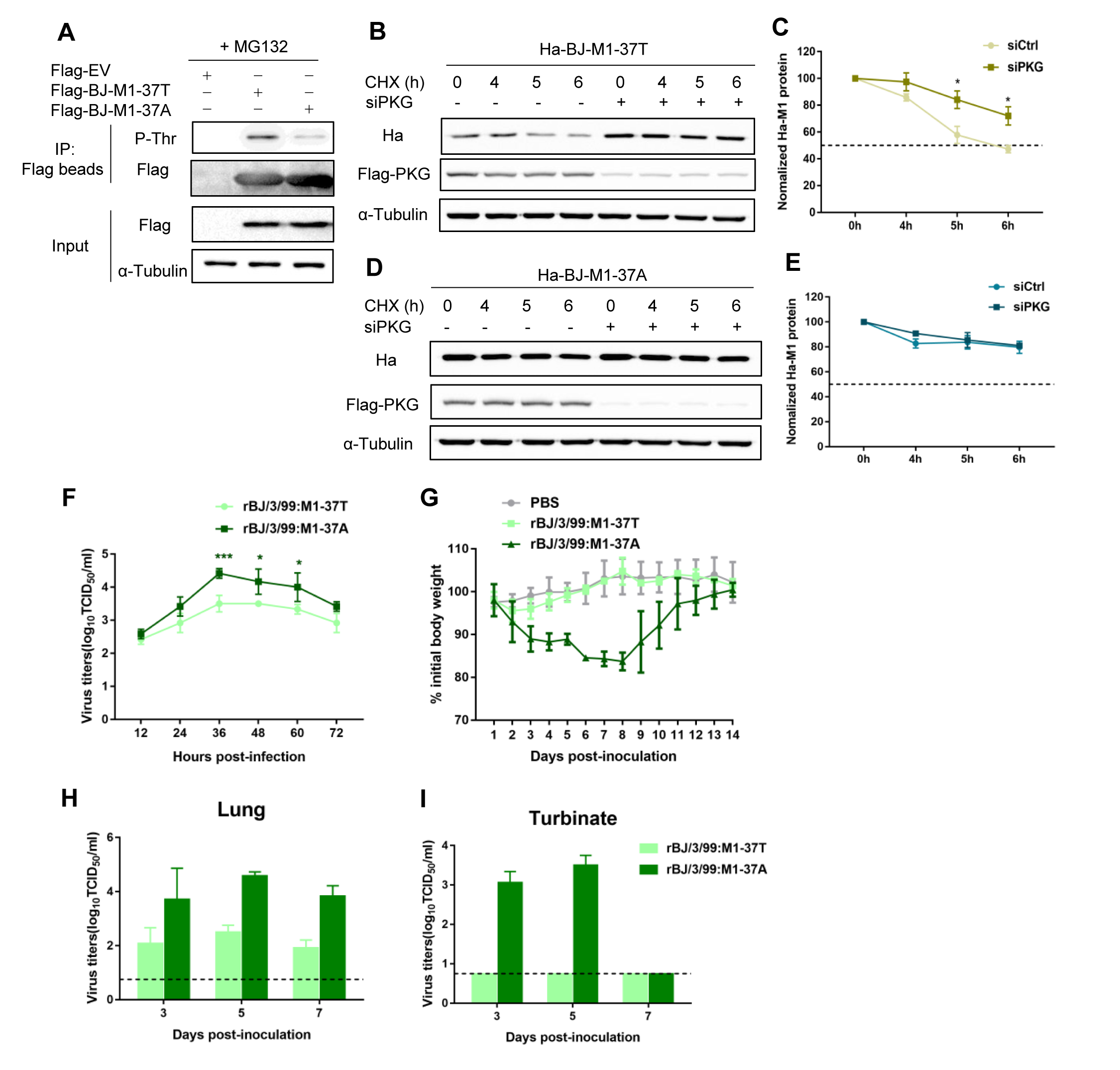

Supplement: S5 Fig — (A) Immunoblotting (IB) analysis of the threonine phosphorylation levels of M1 of BJ/3/99 virus. HEK293T cells transfected with Flag-tagged BJ/3/99 M1 (37T or 37A) expression plasmids or empty vector (Flag-EV) plasmid for 24 h, followed by MG132 (20 μM) treatment for 6 h, were immunoprecipitated with Flag beads. Threonine phosphorylation levels of M1 were detected by an anti-phosphothreonine antibody. α-Tubulin was used as a loading control. (B-E) Western blotting (WB) analysis of the half-life of BJ/3/99 M1 protein in control or PKG-silenced A549Flag-PKG cells. Control or PKG-silenced A549Flag-PKG cells were transfected with Ha-tagged BJ-M1-37T (B) or BJ-M1-37A (D) plasmid for 24 h, then treated with CHX for the indicated time. WB was performed to analyze the expression levels of Ha-M1 and Flag-PKG. Data were quantified as the ratio of Ha-M1 to α-Tubulin and were displayed in respective graphs (C, E), and the data represent the mean ± SD pooled from three independent experiments. Statistical significance was based on two-way ANOVA (**P<0.01; ***P<0.001). All WB data are representative of three independent experiments showing similar results. (F) Virus output of rBJ/3/99:M1-37T and rBJ/3/99:M1-37A viruses from infected A549 cells. A549 cells were infected at an MOI of 0.4. Virus titers were determined from supernatants collected at the indicated time points. Data are presented as the mean ± SD of three independent experiments. Statistical significance was based on two-way ANOVA (*P<0.05; **P<0.01; ***P<0.001). (G) Body weight changes in mice over a 14-day period. Six-week-old female BALB/c mice (n = 5 per group in one independent experiment) were individually inoculated with 106 TCID50 of rBJ/3/99:M1-37T or rBJ/3/99:M1-37A or were mock infected with PBS. Each data point represents the mean ± SD and is representative of three independent experiments. Statistical significance was based on two-way ANOVA (*P<0.05; **P<0.01). (H, I) Virus titers recovered from mouse lung [file ppat.1010645.s005.tif]

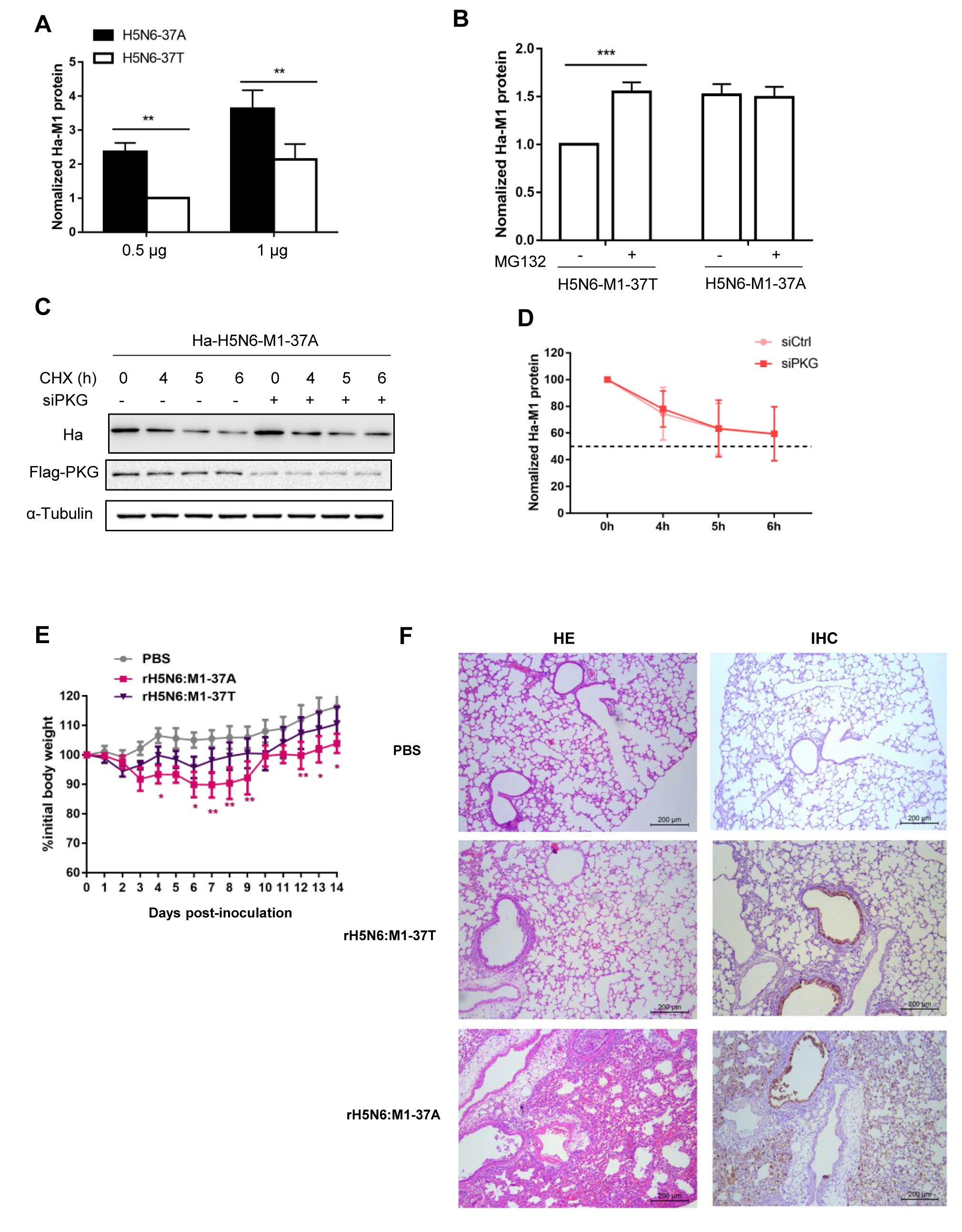

Supplement: S6 Fig — (A, B) Densitometry analysis of the data presented in Fig 6A and Fig 6F, respectively. The data represent the mean ± SD pooled from three independent experiments. Statistical significance was based on t-tests (**P<0.01; ***P<0.001). (C, D) Western blotting (WB) analysis of the half-life of M1 protein in control or PKG-silenced A549Flag-PKG cells. Control or PKG-silenced A549Flag-PKG cells were transfected with Ha-tagged H5N6-M1-37A plasmid for 24 h and then treated with CHX (50 μg/mL) for the indicated time. WB was performed to analyze the expression levels of Ha-M1 and Flag-PKG. Data are quantified as the ratio of Ha-M1 to α-Tubulin and are displayed in graph (D). The data represent the mean ± SD pooled from three independent experiments. Statistical significance was based on two-way ANOVA. The WB data are representative of three independent experiments showing similar results. (E) Bodyweight changes in mice over a 14-day period. Six-week-old female BALB/c mice (n = 5 per group in one independent experiment) were individually inoculated with 106 TCID50 of rH5N6:M1-37T or rH5N6:M1-37A viruses or were mock infected with PBS. Each data point represents the mean ± SD and is representative of three independent experiments. Statistical significance was based on two-way ANOVA (*P<0.05; **P<0.01). (F) Representative HE and IHC examination of lung sections from three BALB/c mice infected with 106 TCID50 of rH5N6:M1-37T and rH5N6:M1-37A viruses at 3 dpi. Scale bar, 200 μm. (TIF) [file ppat.1010645.s006.tif]

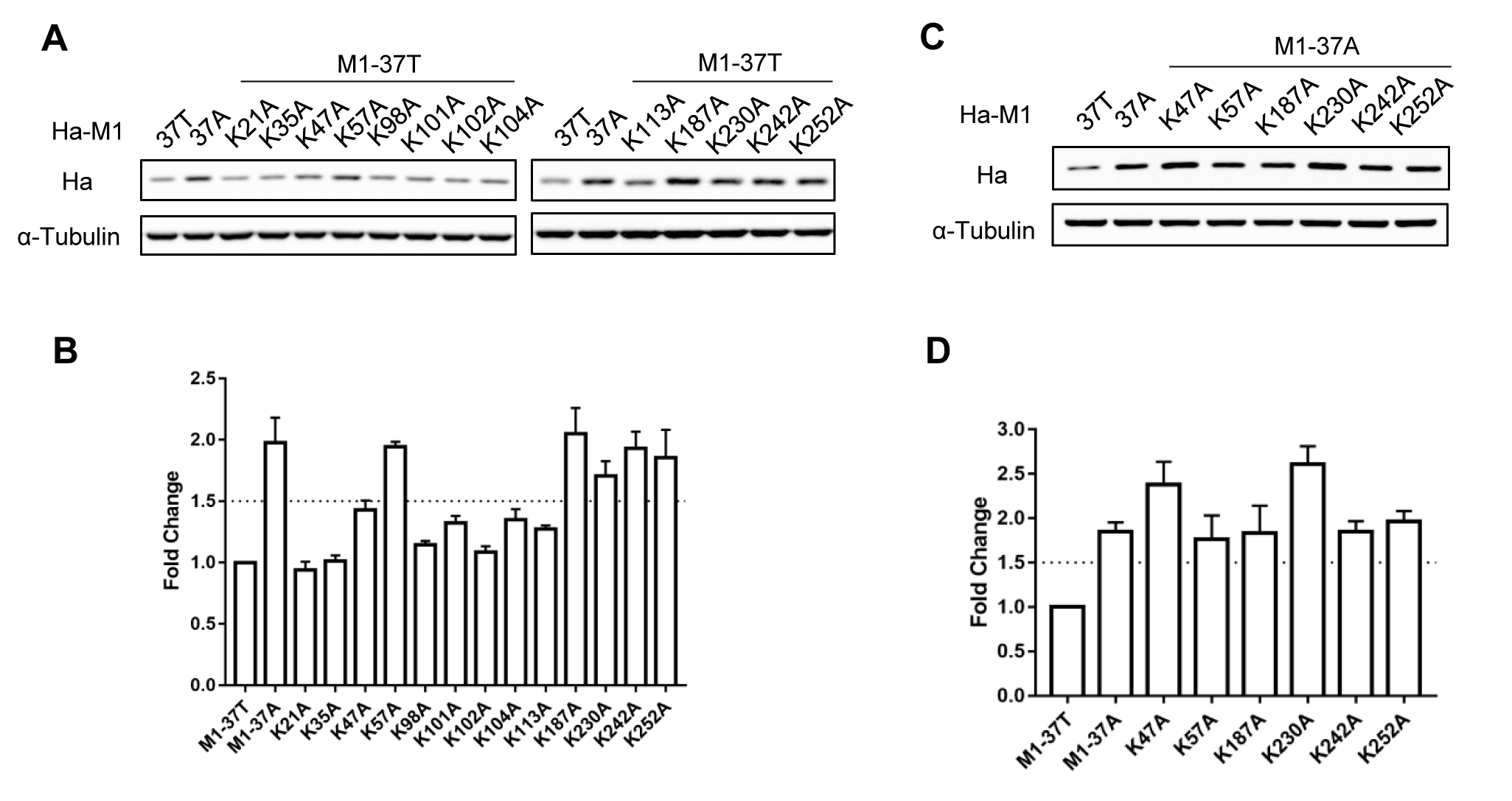

Supplement: S7 Fig — (A) A549 cells were transfected to express the Ha-tagged M1-37T, M1-37A, or indicated K mutants of M1-37T, respectively. Western blotting (WB) was used to detect the M1 expression levels using an anti-Ha antibody. (B) Densitometry analysis of the data presented in (A). (C) Mutations that conferred significantly increased M1-37T expression in S7A Fig were introduced into M1-37A. HEK293T cells were transfected to express the Ha-tagged M1-37T, M1-37A, or indicated K mutants of M1-37A, respectively. WB was used to detect the expression levels of M1 in A549 cells using an anti-Ha antibody. (D) Densitometry analysis of the data presented in (C). α-Tubulin was used as a loading control. All data represent the mean ± SD pooled from three independent experiments. (TIF) [file ppat.1010645.s007.tif]

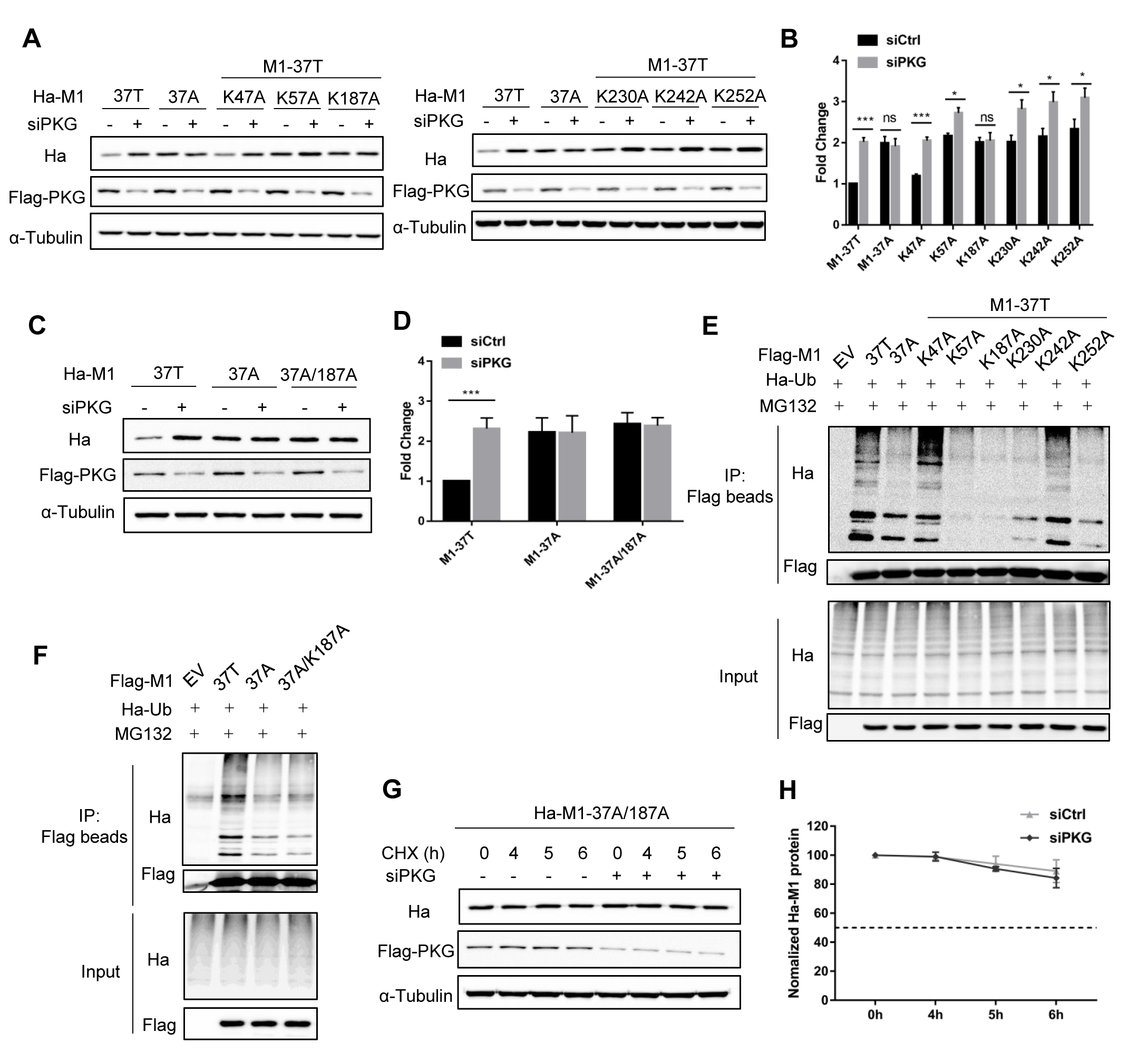

Supplement: S8 Fig — (A) Western blotting (WB) analysis of Ha-M1 and Flag-PKG in control or PKG-silenced A549Flag-PKG cells transfected with plasmids for Ha-tagged M1-37T, M1-37A, or the indicated mutants of M1-37T. (B) Densitometry analysis of the data presented in (A). (C) WB analysis of Ha-M1 and Flag-PKG in control or PKG-silenced A549Flag-PKG cells transfected with plasmids for Ha-tagged M1-37T, M1-37A, or M1-37A/187A. α-Tubulin was used as a loading control. (D) Densitometry analysis of the data presented in (C). All data represent the mean ± SD pooled from three independent experiments. Statistical significance was based on t-tests (*P<0.05; **P<0.01; ***P<0.001). (E, F) Ubiquitination analysis of M1-37T mutants (E) or M1-37A mutants (F). Ha-tagged ubiquitin (Ha-Ub) plasmid was co-transfected with the indicated Flag-tagged M1 plasmid or empty vector (EV) plasmid into HEK293T cells for 24 h, followed by MG132 (20 μM) treatment for 6 h. Ubiquitinated proteins were then analyzed by WB. (G) WB analysis of the half-life of M1-37A/187A protein in control or PKG-silenced A549Flag-PKG cells. Control or PKG-silenced A549Flag-PKG cells were transfected with Ha-tagged M1-37A/187A plasmids for 24 h and then treated with CHX for the indicated time. WB was performed to analyze the expression levels of Ha-M1 and Flag-PKG. Densitometry analysis of the data presented in Fig G is displayed in graph (H), and the data represent the mean ± SD pooled from three independent experiments. Statistical significance was based on two-way ANOVA. All WB data are representative of three independent experiments showing similar results. (TIF) [file ppat.1010645.s008.tif]

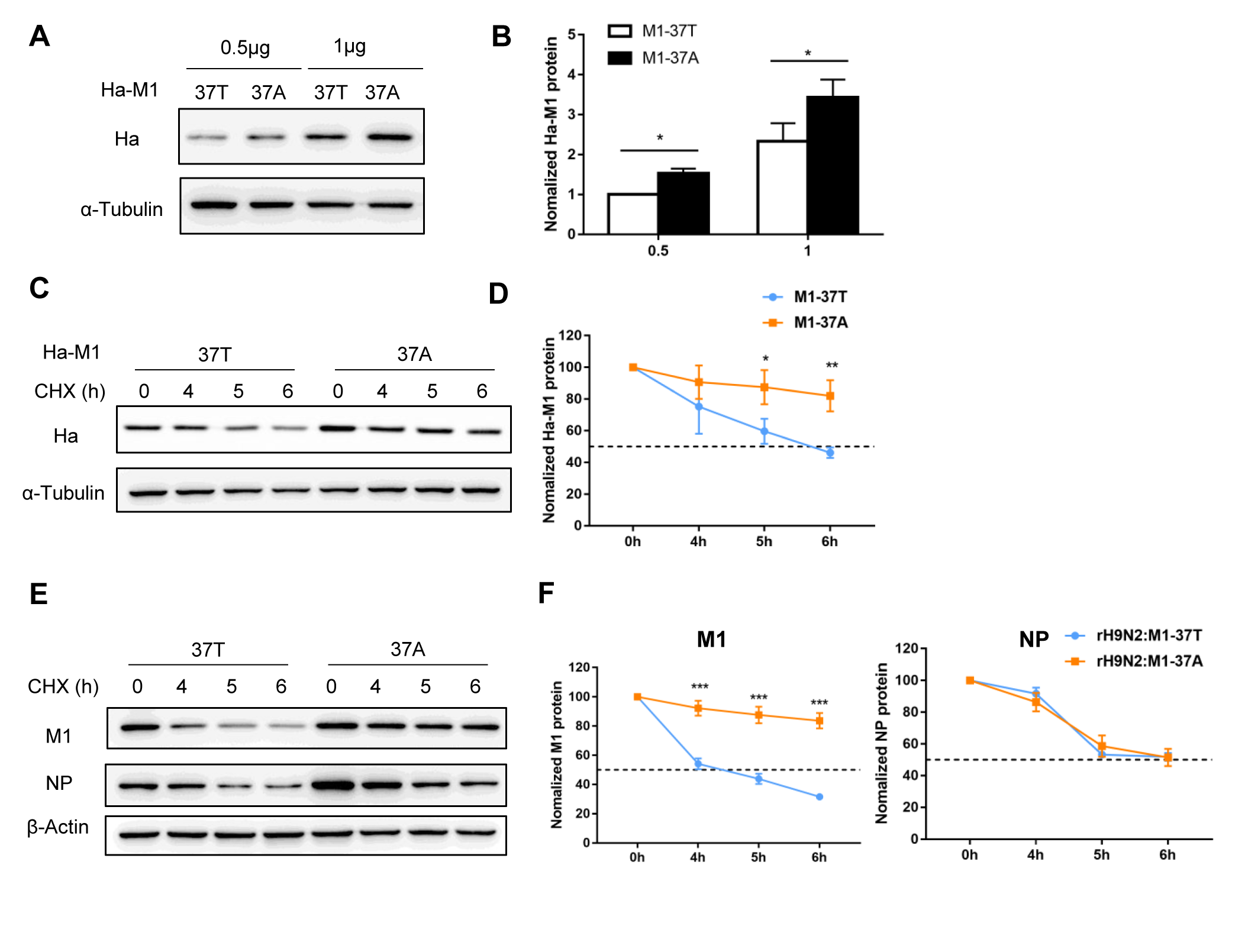

Supplement: S9 Fig — (A) The M1 protein abundance levels at different transfection doses in DF1 cells. DF1 cells were transfected with the indicated amounts of plasmids containing Ha-tagged M1 (37T or 37A). The Ha-M1 protein was determined by immunoblotting analysis with α-Tubulin used as a loading control. Densitometry analysis of the data presented in (A) are displayed in graph (B), and the data represent the mean ± SD pooled from three independent experiments. Statistical significance was based on t-tests (*P<0.05). (C-F) Protein degradation assay of M1 protein in DF1 cells. DF1 cells were transfected with Ha-tagged M1 (37T or 37A) expression plasmids for 24 h (C), or were infected with rH9N2:M1-37T or rH9N2:M1-37A for 24 h (E), followed by CHX (50 μg/mL) treatment over the indicated time course. NP protein was used as a viral protein control. The half-life of M1 or NP proteins (normalized to α-Tubulin) as determined by western blotting is displayed in the respective graphs. Densitometry analysis of the data presented in (C, E) are displayed in respective graphs (D, F), and the data represent the mean ± SD pooled from three independent experiments. Statistical significance was based on two-way ANOVA (*P<0.05; **P<0.01; ***P<0.001). (TIF) [file ppat.1010645.s009.tif]

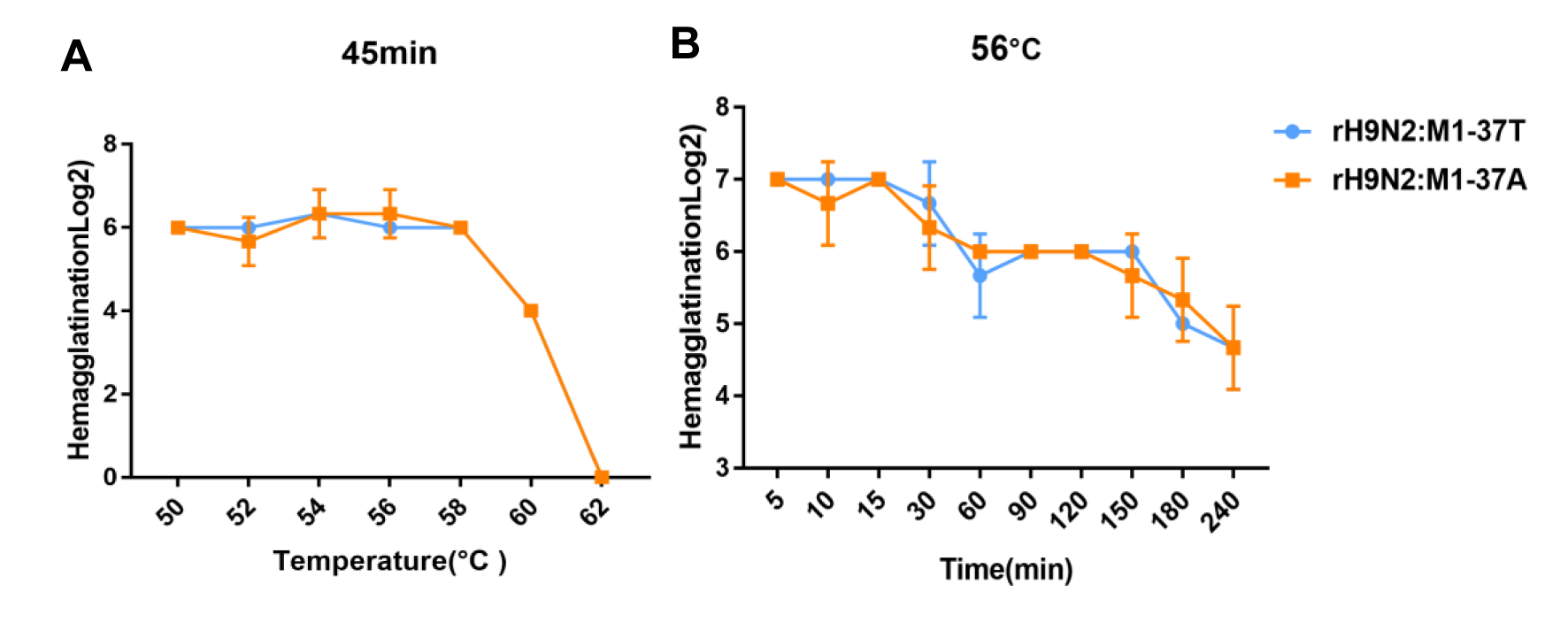

Supplement: S10 Fig — Viruses were adjusted to 7 log2 HA units. The thermal stability was assessed by examining the ability of each virus to hemagglutinate chicken erythrocytes after incubation at the indicated temperatures for 45 min (A) or at 56°C for the time course up to 240 minutes (B). The data are representative of three independent experiments. (TIF) [file ppat.1010645.s010.tif]

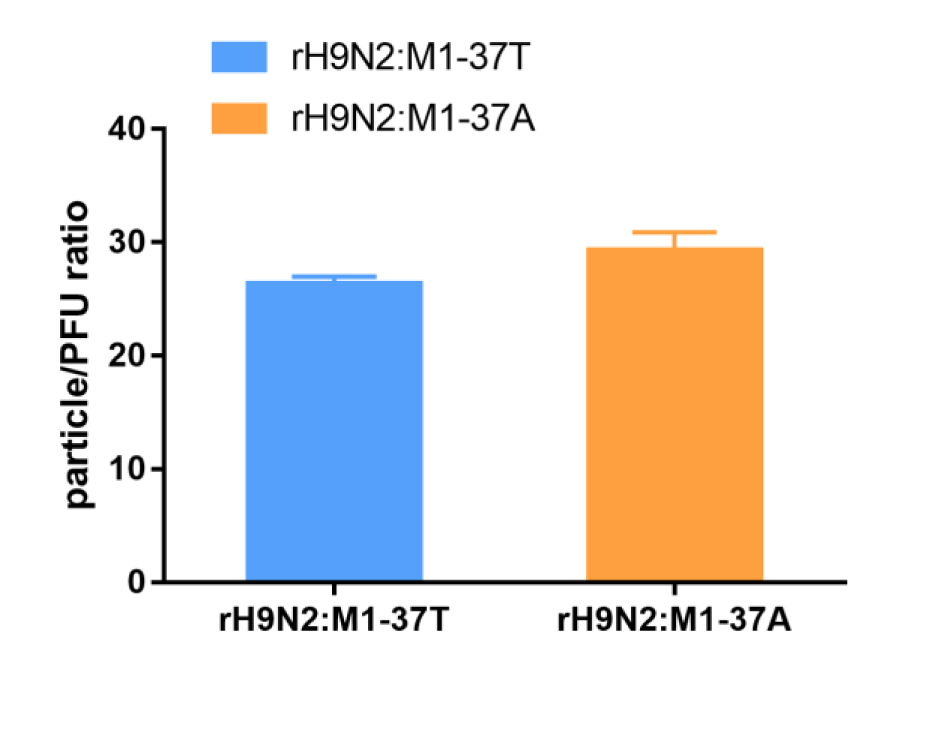

Supplement: S11 Fig — The viral particle to PFU ratio was determined as previously described [53]. Briefly, RNA was extracted and purified from each culture supernatant, and the viral RNA copy number in the supernatant was determined by qRT-PCR and regarded as the number of virus particles. A virus titer in PFU of each culture supernatant was also determined by plaque assay, and particle-to-PFU ratio was calculated. The particle to PFU ratio of the rH9N2:M1-37T and rH9N2:M1-37A are 26.3±0.64 and 29.3±1.60, respectively. (TIF) [file ppat.1010645.s011.tif]

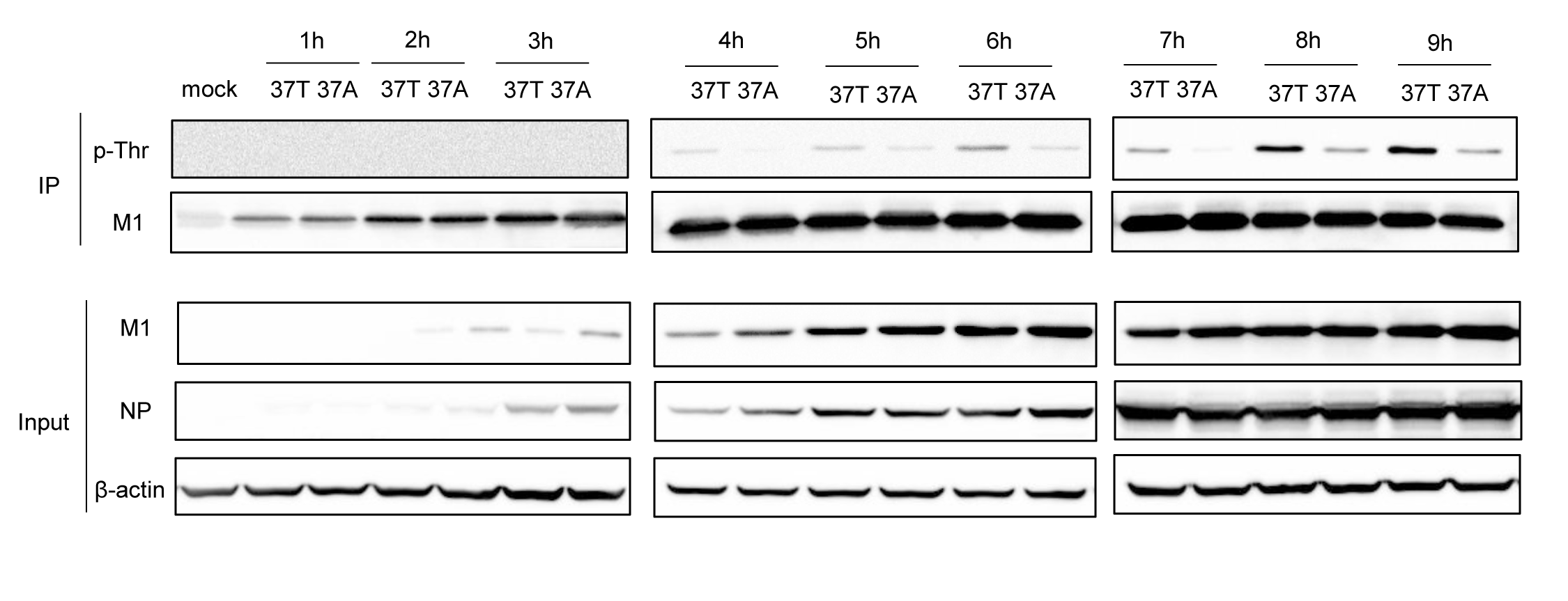

Supplement: S12 Fig — A549 cells were infected with the rH9N2:M1-37T or rH9N2:M1-37A H9N2 viruses at an MOI of 10 for 1–9 h. Cells were then harvested and lysed at the indicated times post infection, and the threonine phosphorylation levels of M1 were detected by anti-phosphothreonine antibody. Western blotting analyzed the expression levels of M1 and NP, and β-Actin was used as loading control. (TIF) [file ppat.1010645.s012.tif]

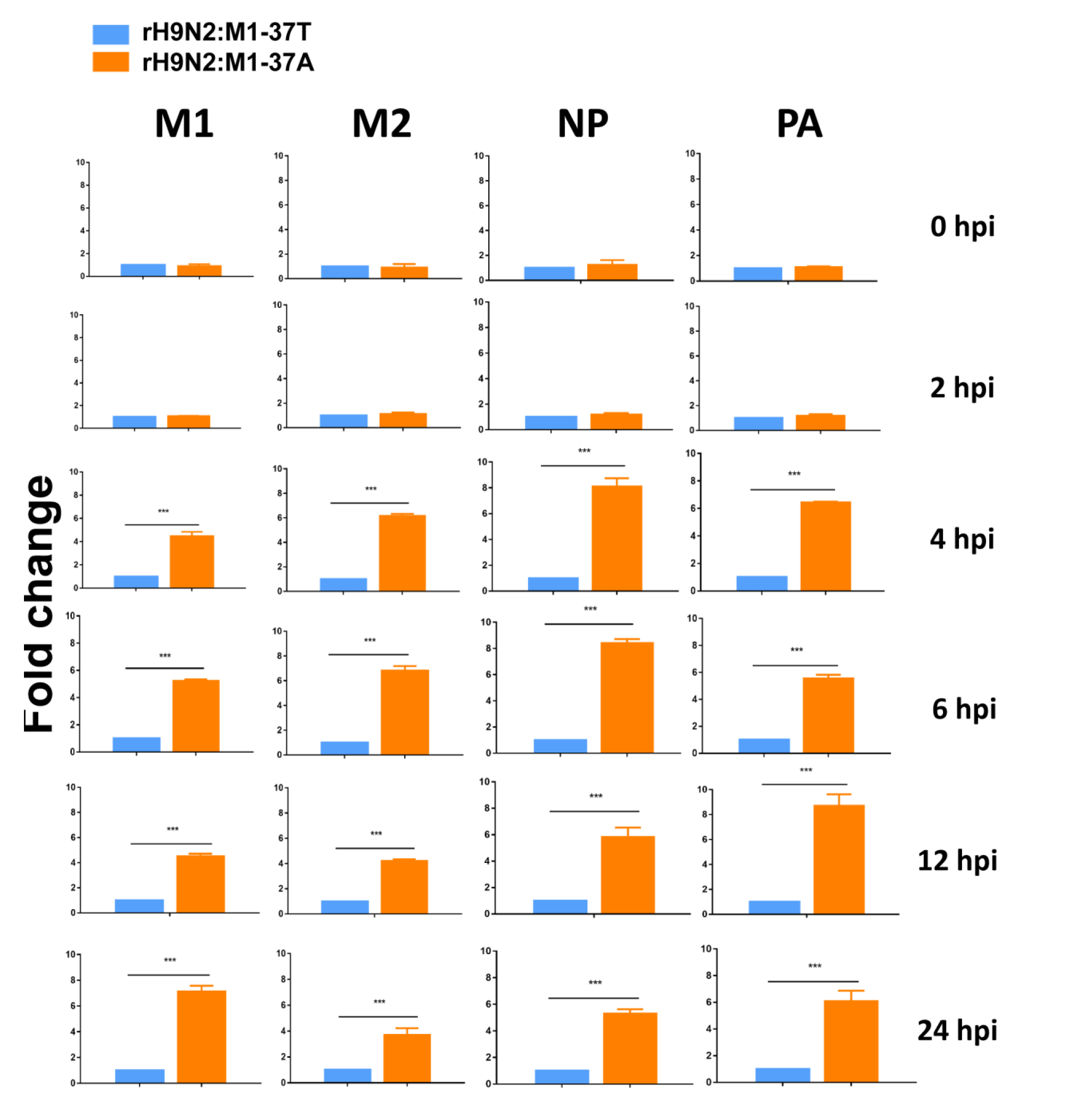

Supplement: S13 Fig — A549 cells were infected with the indicated H9N2 viruses at an MOI of 1 for 0, 2, 4, 6 and 12 h or at an MOI of 0.2 for 24 h. The mRNA expression levels are presented as fold changes relative to the values for the rH9N2:M1-37T virus. Data are presented as mean ± standard deviations of results from three independent experiments. Statistical significance was based on two-way ANOVA (***P<0.001). (TIF) [file ppat.1010645.s013.tif]

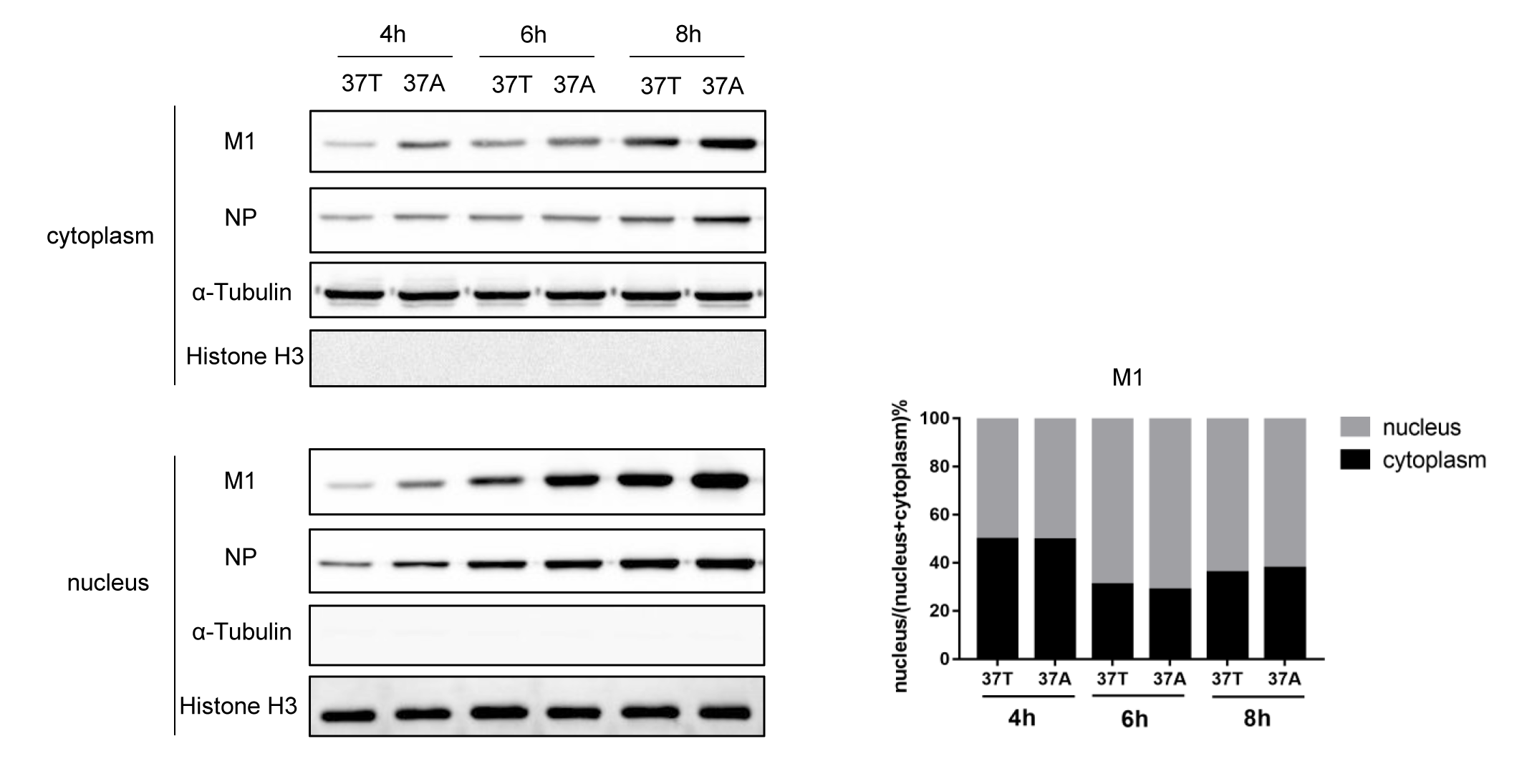

Supplement: S14 Fig — A549 cells were infected at an MOI of 2 with rH9N2:M1-37T or rH9N2:M1-37A. Cells were harvested at 4, 6, 8 hpi. The nuclear and cytoplasmic fractions were prepared using a Nuclear and Cytoplasmic Protein Extraction Kit (Beyotime Biotechnology, Shanghai, China) according to the manufacturer’s instructions. Lysates were analyzed by immunoblotting with antibodies to M1, NP, α-Tubulin and Histone H3. These results represent three independent experiments. (TIF) [file ppat.1010645.s014.tif]

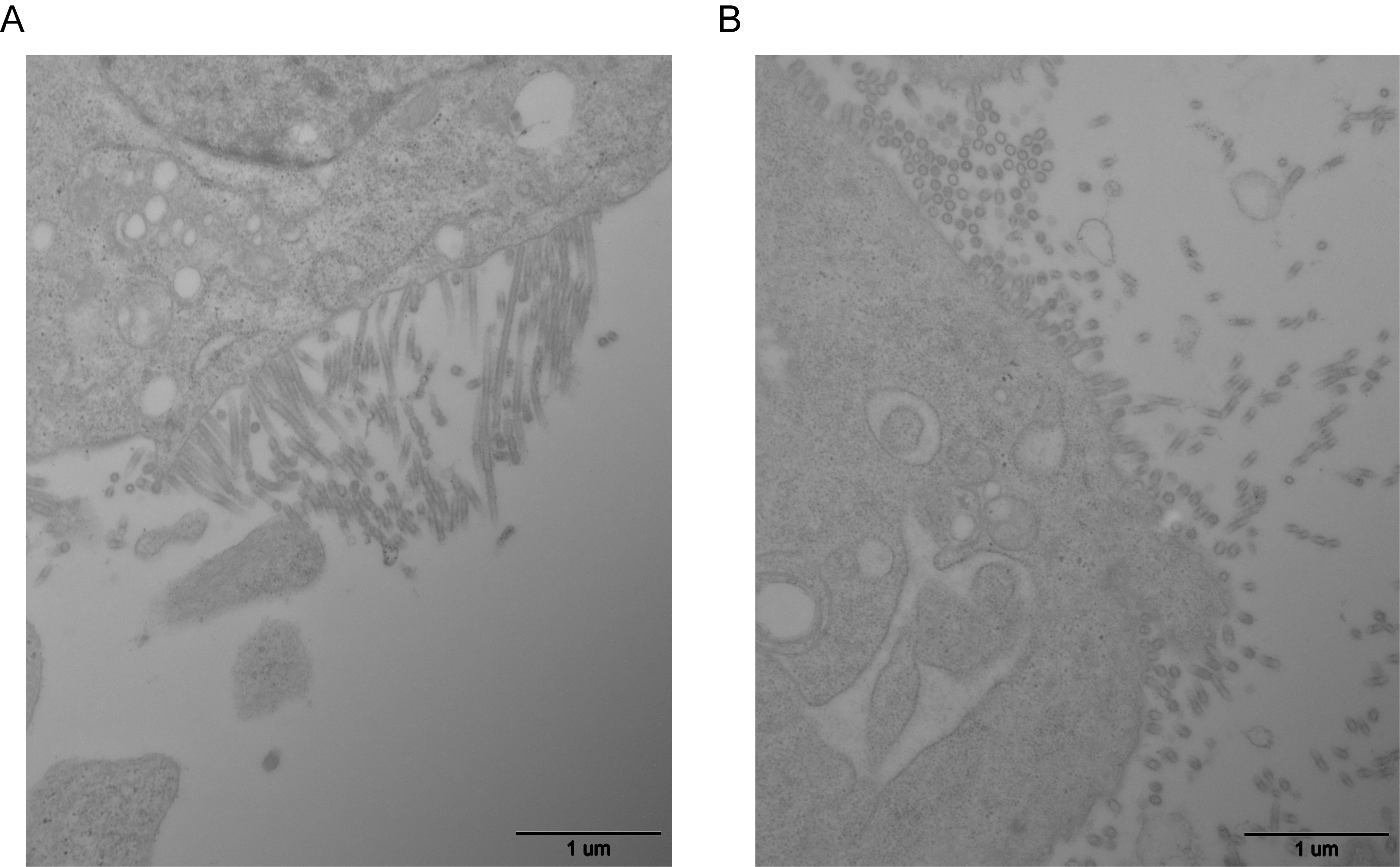

Supplement: S15 Fig — For imaging of virions, samples were prepared as previously described [22]. Cells were infected at an MOI of 3.0 for 15 h. rH9N2:M1-37T progeny were mainly filamentous, and rH9N2:M1-37A particles were primarily spherical/ovoid. (TIF) [file ppat.1010645.s015.tif]
